# LINC01852 inhibits the tumorigenesis and chemoresistance in colorectal cancer by suppressing SRSF5-mediated alternative splicing of PKM

**DOI:** 10.1186/s12943-024-01939-7

**Published:** 2024-01-24

**Authors:** Zehua Bian, Fan Yang, Peiwen Xu, Ge Gao, Chunyu Yang, Yulin Cao, Surui Yao, Xue Wang, Yuan Yin, Bojian Fei, Zhaohui Huang

**Affiliations:** 1 Wuxi Cancer Institute, Affiliated Hospital of Jiangnan University, 200 Hui He Road, Wuxi, Jiangsu 214062 China; 2 Laboratory of Cancer Epigenetics, Wuxi School of Medicine, Jiangnan University, Wuxi, Jiangsu 214122 China; 3 Department of General Surgery, Affiliated Hospital of Jiangnan University, Wuxi, Jiangsu 214062 China

**Keywords:** Colorectal cancer, lncRNA, Chemoresistance, Aerobic glycolysis, Alternative splicing

## Abstract

**Background:**

Colorectal cancer (CRC) is a major cause of cancer-related deaths worldwide, and chemoresistance is a major obstacle in its treatment. Despite advances in therapy, the molecular mechanism underlying chemoresistance in CRC is not fully understood. Recent studies have implicated the key roles of long noncoding RNAs (lncRNAs) in the regulation of CRC chemoresistance.

**Methods:**

In this study, we investigated the role of the lncRNA LINC01852 in CRC chemoresistance. LINC01852 expression was evaluated in multiple CRC cohorts using quantitative reverse transcription PCR. We conducted in vitro and in vivo functional experiments using cell culture and mouse models. RNA pull-down, RNA immunoprecipitation, chromatin immunoprecipitation, and dual luciferase assays were used to investigate the molecular mechanism of LINC01852 in CRC.

**Results:**

Our findings revealed that a lncRNA with tumor-inhibiting properties, LINC01852, was downregulated in CRC and inhibited cell proliferation and chemoresistance both in vitro and in vivo. Further mechanistic investigations revealed that LINC01852 increases TRIM72-mediated ubiquitination and degradation of SRSF5, inhibiting SRSF5-mediated alternative splicing of PKM and thereby decreasing the production of PKM2. Overexpression of LINC01852 induces a metabolic switch from aerobic glycolysis to oxidative phosphorylation, which attenuates the chemoresistance of CRC cells by inhibiting PKM2-mediated glycolysis.

**Conclusions:**

Our results demonstrate that LINC01852 plays an important role in repressing CRC malignancy and chemoresistance by regulating SRSF5-mediated alternative splicing of PKM, and that targeting the LINC01852/TRIM72/SRSF5/PKM2 signaling axis may represent a potential therapeutic strategy for CRC.

**Supplementary Information:**

The online version contains supplementary material available at 10.1186/s12943-024-01939-7.

## Background

Colorectal cancer (CRC) ranks third among malignant tumors worldwide in both incidence and mortality [1]. At present, radical surgery is the most common therapeutic approach for CRC. However, the majority of CRC patients are diagnosed at advanced stages of the disease, which means that they have missed the opportunity to benefit from radical surgery. In these patients, chemotherapy or other treatment modalities are typically employed to improve clinical outcomes [2]. Although great progress has been made in the clinical treatment of human cancers, tumor metastasis and recurrence due to chemoresistance remain the main causes of cancer-related deaths. Therefore, revealing the molecular mechanisms underlying chemoresistance is vital for increasing the efficacy of cancer treatments.

Chemoresistance is complicated, and the underling mechanism is not yet fully understood. Long noncoding RNAs (lncRNAs), are noncoding RNA molecules greater than 200 nucleotides in length, and are important regulators of cancer formation and development. LncRNAs have emerged as promising tools for therapeutic intervention and prognostic prediction [3, 4]. Previous studies have shown that several lncRNAs are involved in chemoresistance in various cancer types, including CRC [3, 5–7]. For example, hypoxia-induced LUCAT1 interacts with PTBP1 to facilitate tumor growth and drug resistance by regulating the alternative splicing of downstream DNA damage related genes in CRC [5]. We previously revealed that LINC00152 upregulates the expression of NOTCH1, promoting tumor progression and the resistance of CRC cells to 5-fluorouracil (5-FU) [7]. Additionally, we reported that UCA1 can promote CRC growth and 5-FU resistance by competitively binding to miR-204-5p and upregulating the expression of its target genes [6]. These findings suggest that understanding the molecular mechanisms underlying lncRNA-mediated chemoresistance may provide new insights into the development of more effective cancer treatments.

This study focused on a specific lncRNA called LINC01852 (previously termed ENST00000434223), which is aberrantly expressed in CRC, according to our transcriptome data [8, 9], as well as in several other cancer types [10–12]. We revealed that LINC01852 is downregulated in CRC and that its overexpression inhibits cancer cell growth and chemoresistance. Mechanistically, LINC01852 binds serine/arginine rich splicing factor 5 (SRSF5) and promotes its degradation and suppresses SRSF5-mediated alternative splicing of PKM2, thereby inhibiting aerobic glycolysis, tumor growth and chemoresistance.

## Materials and methods

### Clinical samples

Primary CRC tissues and corresponding adjacent noncancerous tissues (NCTs) were collected at Affiliated Hospital of Jiangnan University. All participants in the study provided written informed consent and voluntarily participated in the experiments. After collection, the tissues were immediately transferred to liquid nitrogen and then stored at -80 °C for preservation until further analyses. The clinical characteristics of the CRC patients are shown in Supplementary Table S1. The study was conducted in accordance with approved guidelines and was approved by the Clinical Research Ethics Committee of Jiangnan University (LS2021102).

### Cell lines

The normal colonic epithelial cell line NCM460 and CRC cell lines (HT29, DLD1, RKO, HCT8 and HCT116) were obtained from the American Type Culture Collection (ATCC). All these cells were cultured at 37 °C with 5% CO_2_ in DMEM supplemented with 10% fetal bovine serum.

### Quantitative reverse transcription PCR (qRT-PCR)

Total RNA was extracted from cells or tissues using RNA isolater Total RNA Extraction Reagent (Vazyme, China) and subjected to a two-step reverse transcription process using a Takara Reverse Transcription Kit (Takara, Japan). Gene expression levels were quantified by qRT-PCR using Ultra SYBR Mixture (CWBIO, China) on a ViiA 7 Real-Time PCR System (Applied Biosystems, USA) with the following thermal cycling program: pre-denaturation for 10 min at 94 °C followed by 40 cycles of denaturation for 15 s at 94 °C and annealing/extension for 1 min at 60 °C/72 °C. The qRT-PCR data were analyzed using the 2^−△△Ct^ method, with ACTB serving as an internal reference. The relevant primer sequences are listed in Supplementary Table S2.

### Cell transfection and generation of stable cell lines

Antisense oligonucleotides (ASOs) targeting LINC01852 were obtained from Guangzhou RiboBio Co., Ltd. SiRNAs targeting SRSF5 or HOXD8 were obtained from Shanghai GenePharma Co., Ltd. The full length LINC01852 sequence was amplified and inserted into the pLenti-EF1aEGFP-F2A-Puro-CMV-MCS vector using the ClonExpress II One Step Cloning Kit (Vazyme). These ASOs were transfected into cells using riboFECT™ CP Reagent (RiboBio, China), and siRNAs and plasmids were transfected into cells using GenMute siRNA & DNA Transfection Reagent (SignaGen, China) according to the manufacturer’s instructions.

To generate CRC cell lines with stable overexpression of LINC01852, the pLenti or pLenti-LINC01852 plasmid was co-transfected with the ps-PAX2 and pMD2G vectors into HEK-293T cells. After 48 h, the viral particles were collected and used to infect CRC cells to establish stable cell lines. After 72 h of infection, puromycin (2 µg/mL; Beyotime, China) was added for selection of stably transduced cell clones. The LINC01852 overexpression efficiency was confirmed by qRT-PCR. The relevant primers used are listed in Supplementary Table S2.

### Cell counting kit 8 (CCK-8) and colony formation assays

For the CCK-8 assay, HCT116 and HCT8 cells were selected for overexpression of LINC01852, while HT29 cells were chosen for knockdown of LINC01852. Cells were seeded into 96-well plates and incubated for 0, 1, 2, 3, or 4 days, respectively. At specific time points, 10 µL of CCK-8 reagent was added to each well of the 96-well plate, and the plate was incubated for 2 h. The absorbance values were then measured at 450 nm using a microplate reader. For the colony formation assay, ~ 800 cells were seeded in each well of 6-well plates, and these plates were incubated at 37 °C for 10–14 days. The cells were fixed with 10% formalin solution and stained with crystal violet solution.

### Apoptosis analysis

For apoptosis detection, cells were stained using an Annexin V-FITC/PI Apoptosis Assay Kit (Vazyme), and analyzed using a BD FACSCanto II (BD, USA).

### Xenograft mouse model

Four-week-old female BALB/c nude mice (Speford, Suzhou) were subcutaneously injected with 2.0 × 10^6^ HCT116 or HCT-8 cells. For the in vivo tumorigenesis experiments, five mice per group were used. In this assay, after the tumors were visible, the animals were treated with the chemotherapeutic drugs 5-FU (HY-90,006, MCE, USA) and oxaliplatin (L-OHP; 61825-94-3, Selleck, USA) every 3 days. A Vernier caliper was used to measure the size of each tumor every 3 days, and the tumor volume was calculated using the formula: V = length × width^2^ × 0.5. All the animal studies were approved by the Experimental Animal Ethics Committee of Jiangnan University (JN. No20220415b0760906 [119]), ensuring that the study was conducted in accordance with ethical guidelines and that the welfare of the animals was properly maintained.

### Fluorescence in situ hybridization (FISH)

The RNA FISH Probe Mix for LINC01852 or 18S rRNA was synthesized and provided by RiboBio. FISH was performed using a FISH kit (RiboBio) according to the manufacturer’s instructions. Finally, images were captured using an Olympus DP80-Cellsens Microscope Imaging System.

### Western blotting (WB)

Total cellular proteins were extracted using RIPA lysis buffer (Beyotime), separated via electrophoresis on a 10% SDS separation gel (YEASEN, China), and transferred to PVDF membranes (Millipore, Germany). The PVDF membranes were then incubated with primary antibodies, including anti-SRSF5 (1:1000; Abcam, UK), anti-HOXD8 (1:1000; Santa Cruz, USA), anti-TRIM72 (1:2000; Proteintech, USA), anti-PKM2 (1:1000; CST, USA), and anti-GAPDH (1:5000; ABclonal, China) antibodies at 4 °C. Subsequently, the membranes were incubated with Peroxidase AffiniPure Goat Anti-Rabbit/Mouse IgG (H + L) (1:10000; Jackson ImmunoResearch, USA) for 1 h at room temperature. The protein bands were visualized with a SuperPico ECL Chemiluminescence Kit (Vazyme) and imaged using a ChemiDoc XRS + Imaging System (Bio-Rad, USA).

### RNA pull-down and mass spectrometry

In brief, biotin-labeled LINC01852 and truncated LINC01852 fragments were transcribed in vitro using Ribo^TM^ RNAmax-T7 (RiboBio). The obtained biotin-labeled RNAs were then incubated with cell lysates overnight at 4 °C. Next, 30 µL of streptavidin magnetic beads (Thermo Fisher) was added to the mixture, which was subsequently incubated for 2 h at room temperature. After thorough washing with NT2 buffer, the proteins bound to the magnetic beads were eluted by adding 1 x SDS loading buffer and incubating at 100 °C for 10 min. The proteins were then analyzed by SDS-PAGE and silver staining. Finally, the bands containing the differentially expressed proteins were excised and analyzed by mass spectrometry (Applied Protein Technology, Shanghai, China). Supplementary Table S2 provides the details of the primers used for in vitro transcription.

### RNA immunoprecipitation (RIP)

RIP assays were performed following the instructions of the EZ-Magna RIP Kit (Millipore, Germany). The cell lysates were mixed with magnetic beads bound to the appropriate antibodies, and the mixtures were incubated at 4 °C overnight. To purify RNA, proteinase K solution was used, and RNA was extracted using RNA isolater Total RNA Extraction Reagent. Finally, quantitative analysis was performed by qRT-PCR.

### Immunohistochemistry (IHC)

The protein levels of SRSF5, PKM2 and HOXD8 were evaluated via IHC. After baking, dewaxing and hydration, the paraffin-embedded tissue sections were subjected to antigen retrieval and blocking. Subsequently, the sections were incubated with anti-SRSF5 (1:100; Abcam), and anti-HOXD8 (1:150; Santa Cruz) antibodies at 4 °C overnight. The next day, the sections were incubated with secondary antibodies, and staining was visualized using the GTVision III Detection System/Mo&Rb (GeneTech, China).

### Chromatin immunoprecipitation (ChIP)

For DNA-protein crosslinking, we harvested cells and fixed them with formaldehyde for 10 min. ChIP assays were then performed according to the instructions provided by Beyotime. To generate DNA fragments of 200–1000 bp, cell lysates were sonicated, and immunoprecipitation was carried out using an anti-HOXD8 antibody (Santa Cruz) or IgG (Beyotime). The resulting chromatin DNA was recovered and analyzed via PCR. Supplementary Table S2 provides the sequences of the primers used.

### Cycloheximide (CHX) and MG132 treatments

After transfection, CRC cells were treated with CHX (HY-12,320, MCE), and subsequently collected at specific time points. Additionally, CRC cells were treated with MG132 (HY-13,259, MCE), and collected 0 and 6 h after MG132 addition. The proteins were then extracted from these cells and analyzed by WB.

### Statistical analyses

Statistical analyses and graphing were carried out using GraphPad Prism 8.0, Adobe Illustrator 2021, and SPSS Statistics 25. Student’s *t*-test was performed to evaluate the differences between two groups. Dunnett’s multiple comparison test was used for comparisons between several experimental groups with the same control group. Tukey’s multiple comparison test was used to compare differences among multiple groups. Differences in survival rates were determined by the Kaplan-Meier method and compared with the log-rank test. *P* < 0.05 indicated a significant difference.

## Results

### LINC01852 is frequently downregulated in CRC and inversely associated with patient survival

**Fig. 1:**
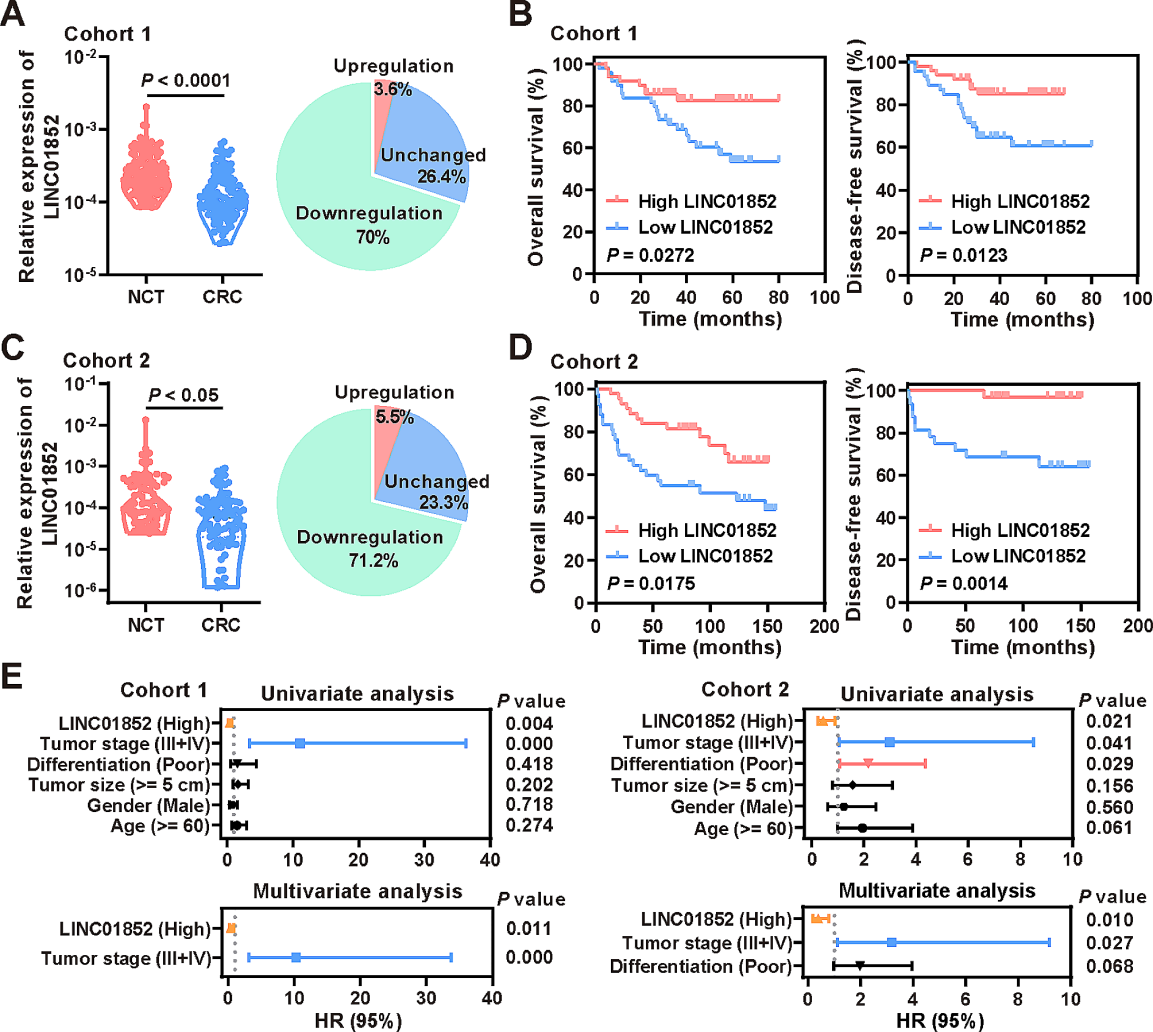
LINC01852 is frequently downregulated in CRC and is inversely associated with patient prognosis. **(A)** The expression of LINC01852 in CRC cohort 1 (110 NCTs and 110 CRC tissues) was measured using qRT-PCR. Changes in LINC01852 expression (more than 1.5-fold) in paired samples are depicted in the pie chart. **(B)** Kaplan-Meier survival analyses with the log-rank tests were performed to investigate the effects of LINC01852 expression on overall survival and disease-free survival in CRC cohort 1. CRC samples were grouped based on the median expression of LINC01852. **(C)** The expression of LINC01852 in CRC cohort 2 (73 NCTs and 85 CRC tissues) was measured using qRT-PCR. **(D)** Kaplan-Meier survival analyses based on LINC01852 expression in CRC cohort 2. **(E)** Univariate and multivariate Cox proportional hazard regression analyses were performed to evaluate the associations between the prognostic factors and overall survival in CRC patients in CRC cohorts 1 and 2

First, we confirmed the expression of LINC01852 in CRC cohort 1 (110 NCTs and 110 CRC tissues) by qRT-PCR and revealed that LINC01852 expression was decreased by more than 1.5-fold in 70% (77/110) of the CRC tissues compared to their paired NCTs (Fig. 1A). Kaplan-Meier survival analyses showed that patients with low LINC01852 expression exhibited poor overall survival (*P* = 0.0272) and disease-free survival (*P* = 0.0123) (Fig. 1B). In addition, these findings were further validated in an independent CRC cohort (73 NCTs and 85 CRC tissues), in which LINC01852 was downregulated in 71.2% (52/73) of the CRC tissues compared to the paired NCTs (Fig. 1C). Survival analyses confirmed that low LINC01852 expression was also associated with poor overall survival (*P* = 0.0175) and disease‒free survival (*P* = 0.0014) in this cohort (Fig. 1D).

Furthermore, univariate and multivariate Cox proportional hazard regression analyses showed that LINC01852 expression was a potential prognostic factor for CRC patients in both cohorts (Fig. 1E). Correlation analyses revealed that LINC01852 expression in CRC tissues was not significantly correlated with tumor stage, differentiation, or tumor size. However, obviously decreased expression of LINC01852 was observed in patients with poor clinical outcomes after adjuvant chemotherapy (Fig. S1). Taken together, these data suggest that LINC01852 plays an important role in CRC tumorigenesis and progression.

### LINC01852 inhibits CRC growth in vitro and in vivo

**Fig. 2:**
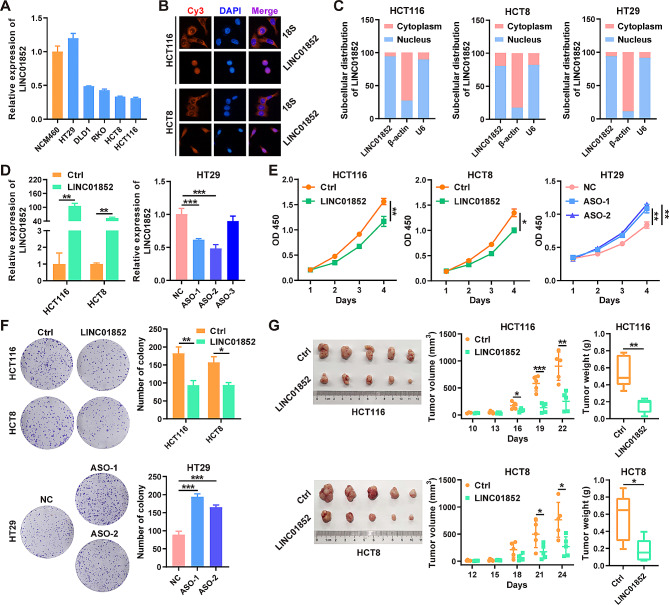
LINC01852 inhibits CRC cell growth in vitro and in vivo. **(A)** qRT-PCR was performed to measure LINC01852 expression levels in different CRC cell lines and NCM460 cells. **(B, C)** The subcellular distribution of LINC01852 in CRC cells was determined using RNA-FISH (B) and qRT-PCR (C). Blue indicates DAPI staining of nuclei, and red (Cy3) indicates LINC01852 or 18S rRNA. **(D)** qRT-PCR validation of LINC01852 expression levels in CRC cells transfected with the LINC01852 overexpression plasmid or the LINC01852-specific ASOs. **(E, F)** The effects of LINC01852 on cell proliferation (E) and colony formation abilities (F). **(G)** The effect of LINC01852 on CRC tumor growth in vivo was evaluated using a xenograft model. Tumor volumes were recorded at the indicated times to construct a growth curve and tumor weights were measured after mouse sacrifice (*n* = 5). *P < 0.05, **P < 0.01, ***P < 0.001

To determine the functional role of LINC01852 in CRC, we first assessed its expression in different CRC cell lines, and found that LINC01852 was downregulated in CRC cell lines compared with the normal colonic epithelium cell line NCM460 (Fig. 2A). Next, we investigated the subcellular distribution of LINC01852 in CRC cells using RNA-FISH and qRT-PCR and revealed that LINC01852 was localized mainly in the nucleus in CRC cells (Fig. 2B and C).

We then carried out gain- and loss-of-function studies of LINC01852 in HCT116, HCT8 and HT29 cells (Fig. 2D). Overexpression of LINC01852 significantly reduced the proliferation and colony formation abilities of CRC cells, whereas silencing LINC01852 expression increased these abilities (Fig. 2E and F). Consistent with these results, in vivo studies showed that ectopic expression of LINC01852 inhibited CRC tumor growth (Fig. 2G). Overall, these findings suggest that LINC01852 exerts a tumor-inhibitory effect in CRC.

### LINC01852 promotes the chemosensitivity of CRC cells

**Fig. 3:**
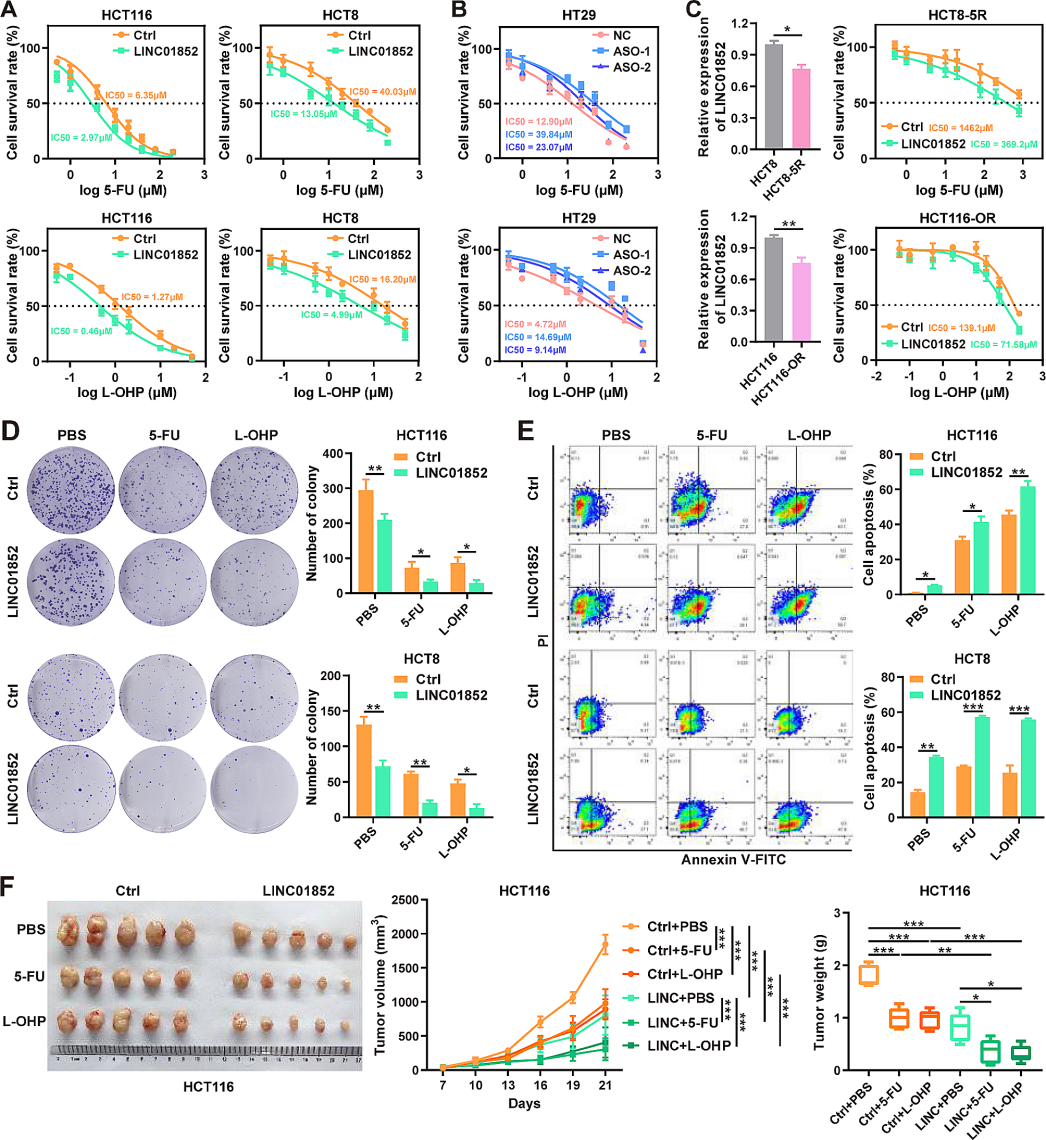
LINC01852 promotes chemosensitivity in CRC cells. **(A, B)** A CCK-8 assay was used to investigate the effects of LINC01852 overexpression and knockdown on the sensitivity of CRC cells to 5-FU and L-OHP. CRC cells were exposed to gradually increasing concentrations of 5-FU and L-OHP for 48 h. **(C)** The LINC01852 expression level and the sensitivity to 5-FU and L-OHP were measured in HCT8-5R and HCT116-OR cells by qRT-PCR and CCK-8 assays, respectively. **(D, E)** The effects of LINC01852 overexpression on the colony formation (D) and apoptosis (E) of CRC cells treated with 5-FU (10 µM for HCT116; 40 µM for HCT8) or L-OHP (2 µM for HCT116; 8 µM for HCT8) for 14 days (colony formation, D) or 48 h (apoptosis, E). **(F)** The effects of LINC01852 overexpression on the in vivo tumorigenicity and chemosensitivity of CRC cells. Nude mice (*n* = 5) were injected with 2 × 10^6^ LINC01852-overexpressing HCT116 cells. Seven days after tumor implantation, 5-FU (50 mg/kg) and L-OHP (10 mg/kg) were intraperitoneally injected into the mice every 3 days. *P < 0.05, **P < 0.01, ***P < 0.001

The aforementioned analyses revealed that LINC01852 was significantly downregulated in CRC tissues from patients with a poor response to chemotherapy, suggesting that LINC01852 could be related to chemosensitivity in CRC patients. Therefore, we assessed the sensitivities of CRC cells to 5-FU and L-OHP, the two chemotherapeutic drugs most commonly used to treat CRC. The results showed that overexpression of LINC01852 significantly increased, whereas silencing LINC01852 expression strikingly reduced the sensitivity of CRC cells to these chemotherapeutic agents (Fig. 3A and B).

To further verify the effect of LINC01852 on chemoresistance, we used 5-FU-resistant HCT8 (HCT8-5R) cells and L-OHP-resistant HCT116 (HCT116-OR) cells [13]. As expected, the expression of LINC01852 was downregulated in these cells (Fig. 3C). Next, we ectopically expressed LINC01852 in these drug-resistant cells, and exposed them to 5-FU and L-OHP. The results demonstrated that ectopic LINC01852 expression restored the sensitivity of HCT8-5R cells to 5-FU and that of HCT116-OR cells to L-OHP, respectively (Fig. 3C). We also found that overexpression of LINC01852 resulted in a decreased colony formation ability and increased apoptosis rate in CRC cells treated with 5-FU and L-OHP, while silencing of LINC01852 had the opposite effects (Fig. 3D, E and Fig. S2).

To further confirm the in vitro findings and explore potential clinical applications, we evaluated the effect of LINC01852 on chemoresistance using an in vivo mouse model. The xenograft HCT116 tumors overexpressing LINC01852 were significantly more sensitive to 5-FU and L-OHP than were the tumors in the control groups (Fig. 3F). Overall, these results demonstrate that LINC01852 inhibits tumorigenesis and promotes chemosensitivity in CRC cells.

### LINC01852 promotes SRSF5 ubiquitination and degradation by interacting with SRSF5 and TRIM72 in CRC cells

**Fig. 4:**
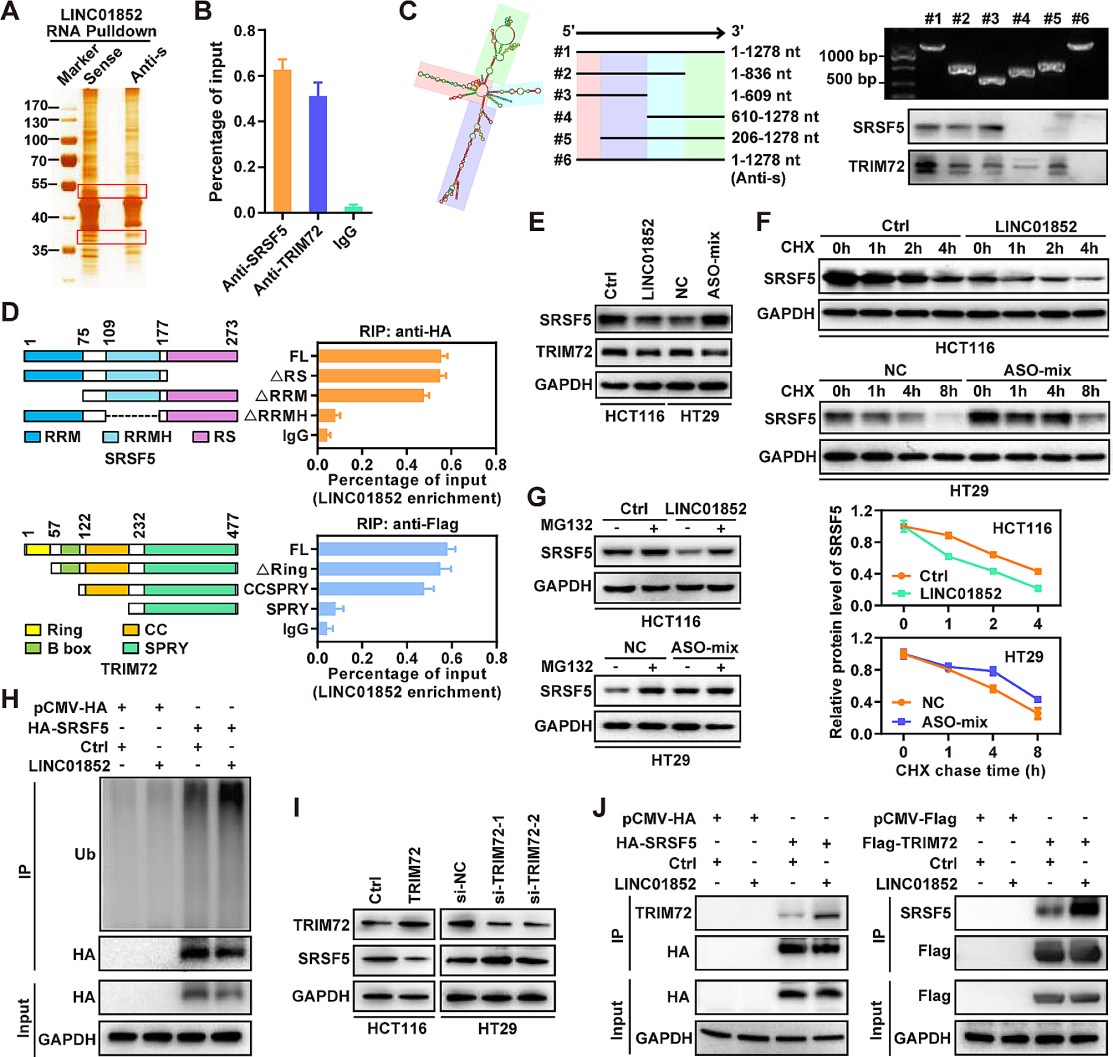
LINC01852 promotes TRIM72-mediated SRSF5 ubiquitination and degradation by interacting with SRSF5 and TRIM72 in CRC cells. **(A)** RNA pull-down followed by silver-staining was performed to determine differential bands for mass spectrometry analyses to identify LINC01852-associated proteins in CRC cells. **(B)** RIP assays showed that SRSF5 and TRIM72 enriched LINC01852 in HCT116 cells. **(C)** Identification of the regions of LINC01852 that mediate the interactions of LINC01852 with SRSF5 and TRIM72. The secondary structure of LINC01852 was predicted via the AnnoLnc2 database. RNA pull-down assays were performed using truncated LINC01852 fragments designed according to the predicted secondary structure. The red area represents the sequence in LINC01852 that binds to SRSF5, and the purple area represents the sequence in LINC01852 that binds to TRIM72. **(D)** RIP assays were performed to identify the regions in SRSF5 and TRIM72 that mediates their interactions with LINC01852 in HCT116 cells transfected with wild-type or truncation mutants of SRSF5 and TRIM72. **(E)** The effects of LINC01852 on the protein levels of SRSF5 and TRIM72 were evaluated by WB. **(F)** Effect of LINC01852 on the endogenous protein level of SRSF5 in CRC cells treated with CHX (50 µg/mL). **(G)** SRSF5 expression in LINC01852-overexpressing and LINC01852-silenced CRC cells. The cells were treated with MG132 (20 µM) for 6 h before harvest. **(H)** Effect of LINC01852 on the ubiquitination of SRSF5. HEK-293T cells were transfected with the indicated vectors for 48 h and subjected to ubiquitination assays. Prior to lysis, the cells were treated with MG132 for 6 h. **(I)** Effect of TRIM72 on the protein level of SRSF5 in CRC cells was detected by WB. **(J)** Co-IP assays were performed to evaluate the effect of LINC01852 on the interaction between SRSF5 and TRIM72 in HEK-293T cells. The cells were treated with MG132 (20 µM) for 6 h before harvest

To identify the potential mechanism mediating the tumor-inhibitory effects of LINC01852, we performed RNA pull-down assays to isolate LINC01852-associated proteins from CRC cells (Fig. 4A). Based on the functional annotation of proteins predicted by mass spectrometry analysis, several proteins were identified as potential LINC01852-associated proteins. The WB results further confirmed the binding of LINC01852 to SRSF5 and TRIM72 using the proteins retrieved from the aforementioned RNA pull-down assays (Fig. S3A). Additionally, RIP assays performed with the antibodies against SRSF5 and TRIM72 showed significant enrichment of LINC01852 (Fig. 4B), indicating that LINC01852 physically associates with SRSF5 and TRIM72.

To determine which region of LINC01852 binds to SRSF5 or TRIM72, we constructed a series of LINC01852 deletion mutants based on the predicted secondary structure of LINC01852 in the AnnoLnc2 database (http://annolnc.gao-lab.org/index.php). RNA fragments transcribed in vitro from these deletion mutant constructs were subjected to RNA pull-down assays. WB analyses revealed that LINC01852 relies on different regions for its binding to SRSF5 and TRIM72. Specifically, deletion of nt 1-205 nearly completely abolished the ability of LINC01852 to bind to SRSF5, while the deletion of nt 206–609 reduced the ability of LINC01852 to bind to TRIM72 (Fig. 4C). To further analyze the binding domains via which SRSF5 and TRIM72 bind to LINC01852, we transiently expressed various deletion mutants of these proteins in HEK-293T cells and performed RIP assays to investigate the enrichment of LINC01852. The results showed that the RRMH domain of SRSF5 and the CC domain of TRIM72 were essential for their respective binding to LINC01852 (Fig. 4D and Fig. S3B, C).

Given that LINC01852 interacts with SRSF5 and TRIM72, we next investigated whether LINC01852 regulates the expression of these targets in CRC cells. The results showed that the protein level of SRSF5 was significantly decreased in LINC01852-overexpressing HCT116 cells and increased in LINC01852-silenced HT29 cells (Fig. 4E), whereas the mRNA level of SRSF5 did not change with modulation of LINC01852 expression (Fig. S4A), suggesting that LINC01852 downregulates SRSF5 protein expression at the posttranscriptional level. However, LINC01852 did not affect either the protein or mRNA level of TRIM72 (Fig. 4E and Fig. S4B). To further investigate the underlying mechanism, we used the protein synthesis inhibitor CHX to evaluate the effect of LINC01852 on the protein stability of SRSF5. The results showed that LINC01852 overexpression markedly reduced the half-life of SRSF5 in HCT116 cells, whereas LINC01852 silencing increased the half-life of SRSF5 in HT29 cells (Fig. 4F). Moreover, treatment with the proteasome inhibitor MG132 reversed the reduced accumulation of endogenous SRSF5 in LINC01852-overexpressing HCT116 cells and inhibited the accumulation of SRSF5 in LINC01852-depleted HT29 cells (Fig. 4G). These data suggest that LINC01852 interferes with SRSF5 degradation via the ubiquitin-proteasome system. Furthermore, the level of ubiquitinated SRSF5 was significantly increased in LINC01852-overexpressing HCT116 cells, indicating that LINC01852 promotes SRSF5 ubiquitination (Fig. 4H).

As LINC01852 can also bind to the E3 ubiquitin ligase TRIM72, we hypothesized that LINC01852 could act as a scaffold to facilitate the binding of TRIM72 to SRSF5, promoting the ubiquitination and degradation of SRSF5. To test this hypothesis, we first examined the effect of TRIM72 on the expression of SRSF5. Overexpression of TRIM72 decreased the protein level of SRSF5 in CRC cells, while silencing of TRIM72 increased it (Fig. 4I). Furthermore, co-IP assays indicated that LINC01852 overexpression significantly increased the interaction between TRIM72 and SRSF5 in CRC cells (Fig. 4J). Notably, ectopic expression of LINC01852 partially restored the inhibitory effect of TRIM72 knockdown on the ubiquitination modification of SRSF5 (Fig. S4C). Together, these data show that LINC01852 decreases the protein stability of SRSF5 by promoting TRIM72-mediated ubiquitination of SRSF5.

### LINC01852 inhibits aerobic glycolysis via SRSF5-dependent alternative splicing of PKM

To investigate the potential molecular mechanism mediating the functions of the LINC01852/SRSF5 axis in CRC, we used RNA-seq combined with gene set enrichment analysis (GSEA) to assess the transcriptomic changes in CRC cells with altered expression of LINC01852 and SRSF5. The results indicated that LINC01852 and SRSF5 potentially regulate multiple tumor-related signaling pathways (Fig. S5A-D). Interestingly, we observed that ectopic expression of LINC01852 impaired the glycolysis pathway, while suppression of LINC01852 activated it (Fig. S5E). Silencing SRSF5 expression induced the activation of the oxidative phosphorylation (OXPHOS) pathway (Fig. S5F), suggesting that the LINC01852/SRSF5 axis regulates energy metabolism, which plays an important role in the occurrence and development of CRC.

**Fig. 5:**
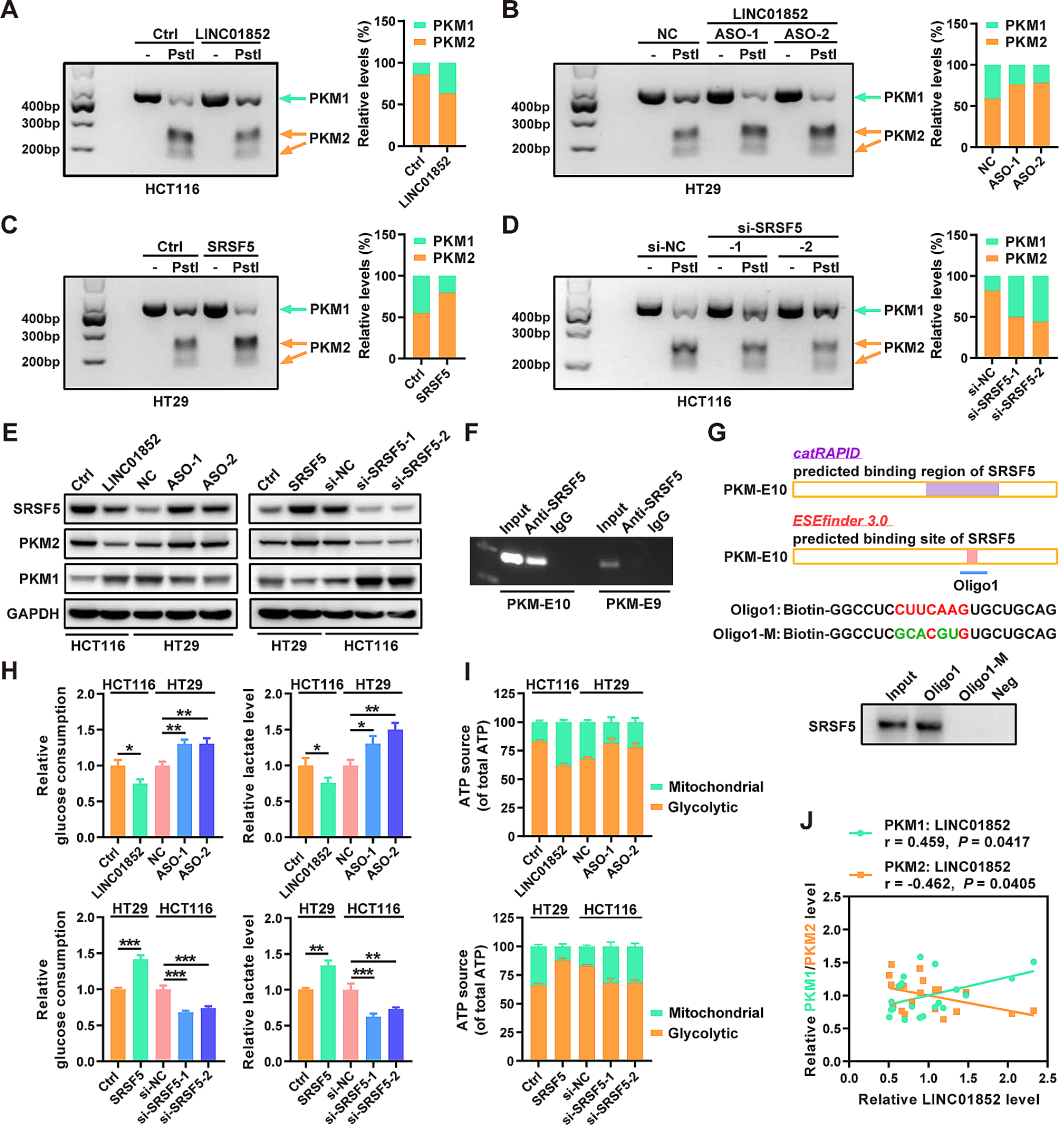
LINC01852 inhibits aerobic glycolysis through SRSF5-dependent alternative splicing of PKM. **(A-D)** PKM splicing assays were performed to measure the relative mRNA levels of PKM1 and PKM2 in CRC cells transfected with LINC01852 overexpression plasmid (A), LINC01852-ASOs (B), SRSF5 overexpression plasmid (C) or SRSF5 siRNAs (D). **(E)** WB was performed to examine the effects of LINC01852 and SRSF5 on the protein levels of PKM1 and PKM2 in CRC cells. **(F)** RIP assays indicated that SRSF5 directly binds to the flanking region of PKM E10. **(G)** RNA pull-down assays revealed that SRSF5 strongly binds to a region in PKM E10 (120–126 bp), which contains an exon splicing enhancer (ESE) for SRSF5. **(H, I)** Effects of LINC01852 and SRSF5 on glucose uptake, lactate production (H), and ATP source (I) in CRC cells. **(J)** The mRNA levels of PKM1 and PKM2 were positively and negatively correlated, respectively, with the LINC01852 level in CRC tissue samples (*n* = 23)

A recent study revealed that SRSF5 can increase the protein expression of PKM2, a key factor regulating the “Warburg effect” in cancer cells [14]. Additionally, SRSF5, a member of the SR-rich protein family, regulates the alternative splicing of several precursor mRNAs (pre-mRNAs). To clarify the relationship between the LINC01852/SRSF5 axis and the alternative splicing of PKM, we examined PKM splicing by RT-PCR followed by restriction digestion to measure the mRNA levels of PKM1 and PKM2, as we previously described [15]. Our results showed that LINC01852 increased, whereas SRSF5 decreased the PKM1/PKM2 ratio (Fig. 5A-D). The WB results confirmed that LINC01852 increased the PKM1 protein levels, while SRSF5 significantly increased PKM2 protein levels in CRC cells (Fig. 5E).

To determine whether SRSF5 is directly involved in regulating the alternative splicing of PKM, RIP assays were conducted in CRC cells. The fragments flanking PKM exon 10 (E10) were enriched in the SRSF5 immunoprecipitate compared to the negative control, suggesting that SRSF5 binds E10 and promotes the production of PKM2 mRNA (Fig. 5F). To identify the detailed binding sites of SRSF5 on PKM E10, we predicted the region in PKM E10 that binds to SRSF5 using catRAPID (http://service.tartaglialab.com/page/catrapid_group), and searched for the exon splicing enhancer (ESE) (a predicted binding site for SRSF5) of PKM E10 using ESE Finder 3.0 (https://esefinder.ahc.umn.edu/cgi-bin/tools/ESE3/esefinder.cgi). RNA pull-down assays revealed that SRSF5 bound with high affinity to the predicted ESE sequence (120–126 bp) in PKM E10, and that mutation of this ESE abolished the ability of SRSF5 to bind to PKM E10 (Fig. 5G).

As the LINC01852/SRSF5 axis can affect the PKM1/PKM2 ratio, we analyzed the impacts of LINC01852 and SRSF5 on glycolysis. Consistent with the above findings, stable overexpression of LINC01852 decreased glucose uptake and lactate production in HCT116 cells, whereas silencing LINC01852 led to the opposite effects in HT29 cells (Fig. 5H). In addition, overexpression of SRSF5 promoted, while silencing SRSF5 expression inhibited glucose uptake and lactate production in CRC cells (Fig. 5H). We further determined the proportions of glycolytic ATP and mitochondrial ATP in these cells to investigate whether the LINC01852/SRSF5 axis affects the shift from glycolysis to OXPHOS. Our data indicated that LINC01852 increased the proportion of mitochondrial ATP, whereas SRSF5 increased the production of ATP via glycolysis (Fig. 5I). Furthermore, we examined the expression levels of the PKM2 and PKM1 isoforms in CRC tissues. The LINC01852 expression level was positively correlated with the PKM1 mRNA level (*r* = 0.459, *P* = 0.0417), and negatively correlated with the PKM2 mRNA level (*r* = − 0.462, *P* = 0.0405) in CRC tissue samples (Fig. 5J). Taken together, our results show that LINC01852 inhibits glycolysis in CRC cells by suppressing SRSF5-mediated PKM splicing.

### LINC01852 increases chemosensitivity by inhibiting SRSF5-mediated PKM2 splicing and thus aerobic glycolysis

The relationships among glycolysis, chemosensitivity, and the LINC01852/SRSF5/PKM2 axis in CRC cells were investigated. We first measured the expression of SRSF5, PKM2 and PKM1 in HCT8-5R and HCT116-OR cells, and the results revealed upregulation of SRSF5 and PKM2, and downregulation of PKM1 in these cells compared to the corresponding parental cells (Fig. S6A). This upregulation was accompanied by increased glucose uptake and lactate production, in these drug-resistant cells (Fig. S6B). To further investigate the role of glycolysis in drug resistance, we inhibited glycolysis in HCT8-5R and HCT116-OR cells using 2-deoxy-D-glucose (2-DG) (Fig. S6C). The results of the CCK-8 assays demonstrated that inhibiting glycolysis partially restored the sensitivity of HCT8-5R cells to 5-FU and that of HCT116-OR cells to L-OHP (Fig. S6D).

**Fig. 6:**
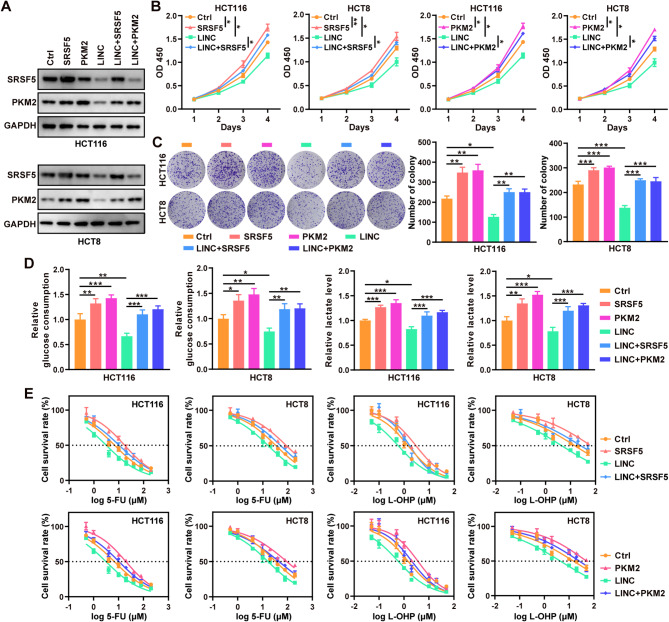
LINC01852 increases the chemosensitivity of CRC cells by inhibiting aerobic glycolysis in an SRSF5/PKM2-dependent manner. **(A)** The relative protein expression levels of SRSF5 and PKM2 in CRC cells transfected with the indicated plasmids were detected by WB. LINC: LINC01852, Ctrl: control vector. **(B, C)** The proliferation (B) and colony formation (C) abilities of HCT116 and HCT8 cells were measured by CCK-8 and colony formation assays, respectively. Cells were transfected with the indicated plasmids as shown in (A). **(D)** Glucose uptake and lactate production were measured in HCT116 and HCT8 cells transfected with the indicated plasmids as shown in (A). **(E)** The sensitivities of CRC cells to 5-FU and L-OHP were assessed by a CCK-8 assay

Based on previous observations linking high glycolytic metabolic activity to drug resistance in CRC cells, we examined whether targeting the LINC01852/SRSF5/PKM2 axis can sensitize CRC cells to 5-FU and L-OHP (Fig. 6A). First, we found that overexpression of SRSF5 and PKM2 partially reversed the tumor suppressive effect of LINC01852 (Fig. 6B and C). Furthermore, the decreases in glucose uptake and lactate production resulting from ectopic expression of LINC01852 were partially reversed by overexpression of either SRSF5 or PKM2 (Fig. 6D). In addition, ectopic expression of LINC01852 significantly sensitized CRC cells to 5-FU and L-OHP (Fig. 6E and Supplementary Table S3), and these effects were obviously blocked by restoring the expression of SRSF5 and PKM2.

These results further support the idea that SRSF5/PKM2 are major targets of LINC01852 and play a crucial role in the resistance of CRC cells to 5-FU and L-OHP.

### The expression of LINC01852 is transcriptionally regulated by HOXD8

**Fig. 7:**
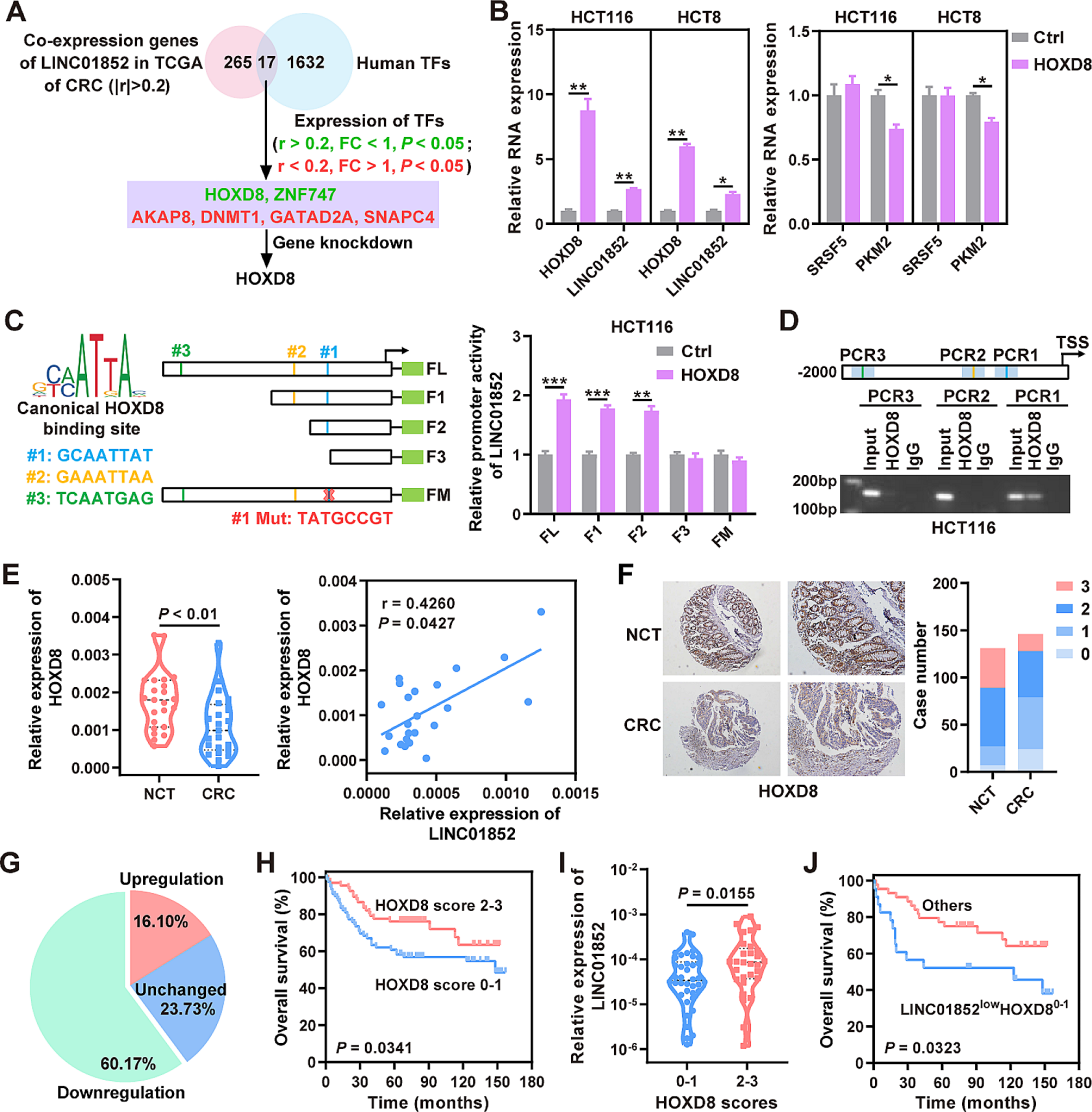
LINC01852 is transcriptionally regulated by HOXD8. **(A)** The flow chart outlines a general approach for screening transcription factors (TFs) that potentially regulate the expression of LINC01852. **(B)** qRT-PCR was used to verify the expression of HOXD8, LINC01852, SRSF5, and PKM2 in HOXD8-overexpressing CRC cells. **(C)** A dual-luciferase reporter assay was used to identify the specific binding site of HOXD8 in the LINC01852 promoter. The left panel shows the HOXD8 binding sites in the LINC01852 promoter; the middle panel shows a schematic representation of the truncated (FL, F1, F2, and F3) and mutated (FM) promoters of LINC01852 in the PGL3-Basic-LINC01852-luc reporter plasmid; the right panel shows the relative luciferase reporter activities of the indicated reporter plasmids. **(D)** ChIP assays were performed to investigate the binding site of HOXD8 in the LINC01852 promoter. **(E)** qRT-PCR was used to measure the expression level of HOXD8 in CRC tissues (*n* = 23), and Pearson correlation analysis was performed to analyze the correlation between HOXD8 mRNA expression and LINC01852 expression. **(F, G)** The protein levels of HOXD8 in CRC tissues (*n* = 146) and NCTs (*n* = 131) were measured via IHC. Representative images of HOXD8 IHC staining (left) and scores (right) in CRC and NCT tissues (F). The pie chart indicates the alteration in HOXD8 expression in paired samples (*n* = 118) (G). **(H)** Kaplan-Meier survival analyses based on the expression of HOXD8 in CRC tissues. **(I)** Violin plots were generated to visualize the distribution of LINC01852 in each group stratified based on the protein expression level of HOXD8. **(J)** Kaplan-Meier survival curves generated based on the expression of LINC01852 and HOXD8 in CRC tissues

Transcription factor (TF) binding is the most common mechanism of gene expression regulation. To identify TFs that regulate LINC01852 expression, we analyzed genes that were co-expressed with LINC01852 in the TCGA database and identified 17 candidate TFs. After filtering for inconsistent expression changes and correlation coefficients, we narrowed the list down to six TFs (Fig. 7A). We then silenced each of these six TFs to explore their effects on LINC01852 expression, and found that only HOXD8 was able to regulate LINC01852 expression (Fig. S7). Moreover, overexpression of HOXD8 in CRC cells increased the LINC01852 level and decreased the PKM2 mRNA level, while the mRNA level of SRSF5 remained unchanged (Fig. 7B). However, the protein levels of both SRSF5 and PKM2 were significantly decreased in HOXD8-overexpressing CRC cells (Fig. S8A).

To identify specific binding sites of HOXD8 in the promoter of LINC01852, we predicted three potential binding sites using the UCSC and JASPAR tools. Through dual-luciferase reporter assays using truncated or mutated LINC01852 reporter constructs, we identified Site 1 as the most likely specific binding site of HOXD8 (Fig. 7C). We then performed a ChIP assay to determine the binding affinity of HOXD8 to the predicated binding sites in the LINC01852 promoter. The results demonstrated that the fragment containing binding Site 1 in the LINC01852 promoter exhibited significant enrichment in HOXD8-ChIPed DNA fragments but not in IgG-ChIPed controls (Fig. 7D).

Subsequently, we examined the mRNA level of HOXD8 in CRC tissues and found that HOXD8 was downregulated in CRC. Pearson correlation analysis revealed that HOXD8 expression was positively correlated with LINC01852 expression in these CRC tissues (*r* = 0.4260, *P* = 0.0427; Fig. 7E) and in CRC tissues from the TCGA database (*r* = 0.2106, *P* < 0.0001; Fig. S9). We also assessed the protein expression of HOXD8 in CRC tissues using IHC and confirmed the downregulation of HOXD8 in CRC (Fig. 7F and G). Survival analyses revealed that low HOXD8 protein expression was closely related to poor survival (*P* = 0.0341, Fig. 7H). Moreover, the protein expression of HOXD8 in human CRC tissues was positively correlated with the expression of LINC01852 (Fig. 7I). The prognosis of CRC patients with low expression of both LINC01852 and HOXD8 was significantly worse than that of CRC patients with other expression patterns (*P* = 0.0323, Fig. 7J).

To determine whether HOXD8 also has an anticarcinogenic effect, we examined cell growth, colony formation ability, chemosensitivity, glucose uptake, and lactate production in HOXD8-overexpressing CRC cells. These cells exhibited a decreased growth rate, impaired colony formation ability, reduced glucose uptake and lactate production. Furthermore, they were more sensitive to 5-FU and L-OHP than were the control cells (Fig. S8B-E). In addition, overexpression of HOXD8 increased the ubiquitination level of SRSF5, which was blocked by TRIM72 knockdown, suggesting that HOXD8 promotes the ubiquitination of SRSF5 via the LINC01852-TRIM72 axis (Fig. S8F). Taken together, these data demonstrate that HOXD8 directly drives the transcription of LINC01852, promoting CRC chemosensitivity.

### SRSF5 and PKM2 expression is upregulated in CRC and negatively correlated with LINC01852 expression

**Fig. 8:**
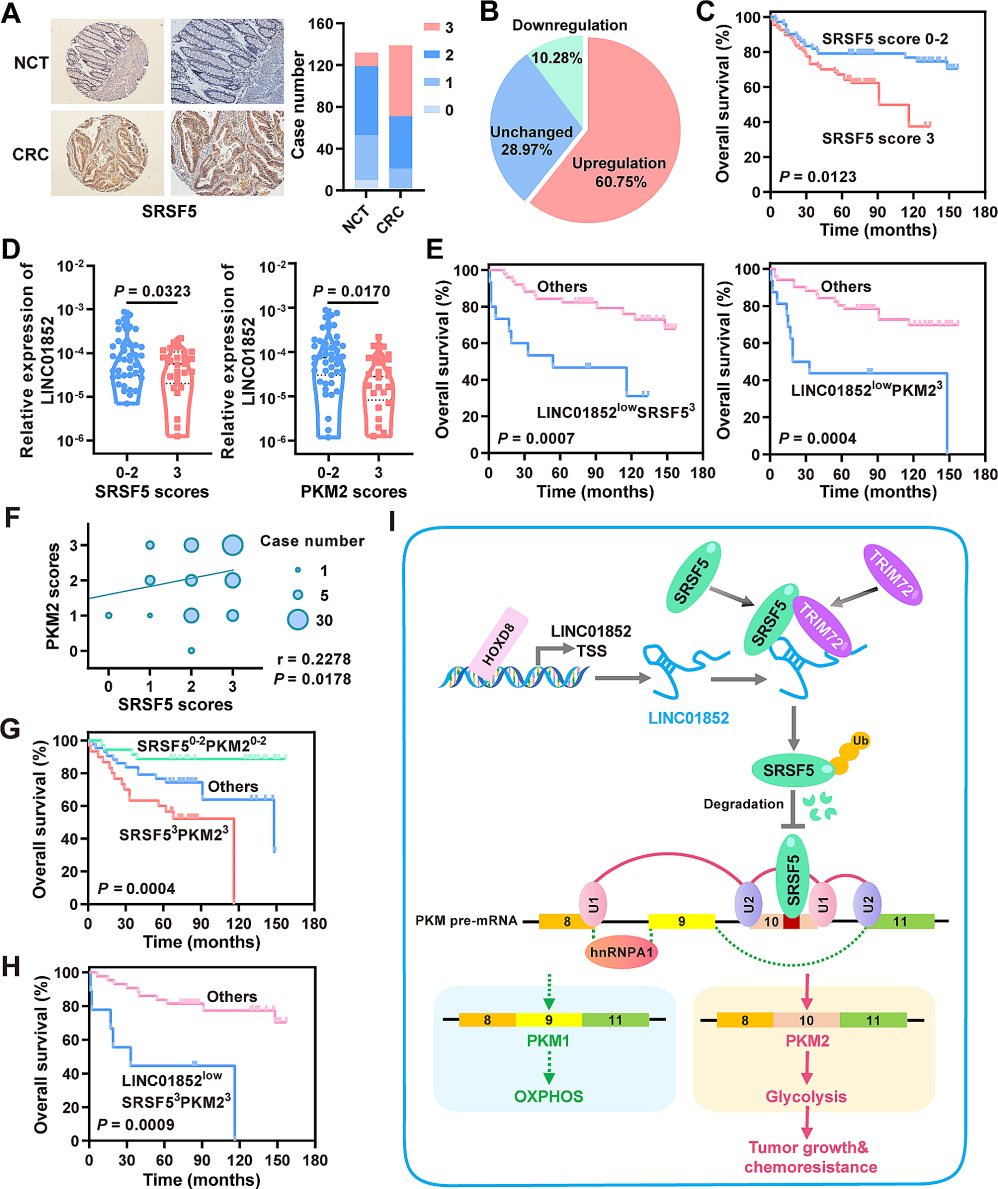
SRSF5 and PKM2 expression is increased in CRC and negatively correlated with LINC01852 expression. **(A)** The protein levels of SRSF5 in CRC tissues (*n* = 139) and NCTs (*n* = 132) were measured via IHC. Representative images of SRSF5 IHC staining (left) and scores (right) are shown. **(B)** Changes in SRSF5 expression in paired samples (*n* = 107) are depicted in the pie chart. **(C)** Kaplan-Meier survival analyses based on the expression of SRSF5 in CRC tissues. **(D)** Violin plots were generated to visualize the distribution of LINC01852 expression in each group stratified based on the protein expression level of SRSF5/PKM2. **(E)** Kaplan-Meier survival curves generated based on the expression of LINC01852/SRSF5 or LINC01852/PKM2 in CRC patients. **(F)** Pearson correlation analyses of SRSF5 and PKM2 protein expression based on IHC staining scores. **(G)** Kaplan-Meier survival curves generated based on the expression of SRSF5 and PKM2 in CRC tissues. **(H)** Kaplan-Meier survival curves based on the expression of LINC01852, SRSF5 and PKM2 in CRC tissues. **(I)** A working model of the mechanism by which LINC01852 suppresses SRSF5-mediated alternative splicing of PKM to affect CRC progression

To investigate the correlations between LINC01852 and its downstream targets, we first assessed the protein expression of SRSF5 in CRC tissues using IHC (Fig. 8A). The results showed that, 60.75% (65/107) of the CRC tissues had increased SRSF5 expression compared with that in the NCTs (Fig. 8B). Survival analyses revealed that high SRSF5 protein expression was closely related to poor survival (*P* = 0.0123, Fig. 8C). Univariate and multivariate Cox proportional hazard regression analyses identified SRSF5 as an independent prognostic factor for CRC (univariate: HR = 2.192, 95% CI: 1.167–4.117, *P* = 0.015; multivariate: HR = 2.192, 95% CI: 1.159–4.147, *P* = 0.016). Furthermore, both SRSF5 and PKM2 protein expression [9] in human CRC tissues was negatively correlated with the expression of LINC01852 (Fig. 8D). The prognosis of CRC patients with low LINC01852 expression and high SRSF5/PKM2 expression was significantly worse than that of CRC patients with other expression patterns (*P* = 0.0007; *P* = 0.0004, Fig. 8E).

A positive correlation between SRSF5 and PKM2 expression was also observed in CRC tissues (Fig. 8F), and the prognosis of CRC patients with high expression of both SRSF5 and PKM2 was significantly worse than that of CRC patients with other expression patterns (*P* = 0.0004, Fig. 8G). Additionally, the prognosis of CRC patients with low LINC01852 expression and high SRSF5 + PKM2 expression was significantly worse than that of CRC patients with other expression patterns (*P* = 0.0009, Fig. 8H). In summary, these findings suggest that SRSF5 and PKM2 are potential downstream targets of LINC01852, and that combination analyses of these three factors have notable value in predicting patient prognosis.

## Discussion

As a major challenge in cancer treatment, chemoresistance is often associated with treatment failure and poor prognosis. LncRNAs have been shown to regulate chemoresistance through multiple mechanisms [3, 5–7, 16, 17]. In this study, we identified that LINC01852 is significantly downregulated in CRC and inhibits CRC cell proliferation, glycolysis, and chemoresistance by promoting TRIM72-mediated SRSF5 degradation and suppressing the alternative splicing of PKM2 (Fig. 8I).

LINC01852, previously named ENST00000434223, has been suggested to be a tumor suppressive lncRNA in several cancer types, including lung cancer [10], gastric cancer [11] and renal cancer [12]. However, its underlying molecular mechanism has not been elucidated. In this study, we revealed that LINC01852 was downregulated in CRC tissues and chemoresistant CRC cells. Functional studies indicated that LINC01852 significantly inhibits CRC growth and chemoresistance, highlighting its tumor suppressive role in CRC.

We and others have reported that lncRNAs can play crucial roles in the regulation of CRC cell proliferation, apoptosis, drug resistance, and metastasis through various mechanisms [6–9, 18–21]. For example, we previously reported that UCA1 and LINC00152 promote CRC chemoresistance via the competing endogenous RNA mechanism [6, 7]. Some lncRNAs can encode short peptides to regulate cell phenotypes, including drug resistance [17]. In addition, Qu et al. reported that cancer-associated fibroblast-derived exosomal DACT3-AS1 promotes tumorigenesis and oxaliplatin resistance in gastric cancer by targeting the miR-181a-5p/SIRT1 axis [16]. In this study, we determined that LINC01852 binds to SRSF5 and regulates its expression. SRSF5 is a member of the serine/arginine (SR) protein family that plays a key role in the regulation of alternative splicing of pre-mRNAs [22]. Abnormal expression of SRSF5 has been reported in a variety of tumors [23, 24]. This study demonstrated that LINC01852 acts as a molecular scaffold to facilitate the interaction between SRSF5 and TRIM72, promoting TRIM72-mediated degradation of SRSF5, and these findings reveal a novel lncRNA-based mechanism that regulates SRSF5 expression and function in cancer cells.

SRSF5 is reported to promote cancer development and progression by regulating the splicing of multiple genes, including ETS1, METTL14, Mcl-1, Cyclin L2, and CCAR1 [22, 23, 25–28]. Specifically, SRSF5 has been shown to promote aerobic glycolysis in CRC cells by increasing glucose consumption and lactate production [26]. In addition, a preliminary study showed that SRSF5 knockdown inhibited glycolysis and cell proliferation by decreasing PKM2 expression in non-small cell lung cancer cells, although the detailed mechanism was unclear [14]. Aerobic glycolysis, a key feature of cancer cells, is closely involved in tumorigenesis, metastasis and drug resistance [25, 29]. Interestingly, RNA-seq analyses revealed that glycolysis is potentially regulated by LINC01852, suggesting that LINC01852 may regulate aerobic glycolysis by inhibiting SRSF5-mediated PKM2 splicing.

The PKM gene generates two different subtypes, PKM1 and PKM2, through selective splicing of PKM pre-mRNA [30]. Although PKM1 and PKM2 differ by only one exon, they have distinct tissue distributions and functions [31]. PKM1 is expressed in normal tissues that require a high energy production capacity, whereas PKM2 is expressed predominantly in tumor tissues to promote aerobic glycolysis [32]. Multiple pathways have been reported to regulate the expression and activity of PKM2 in cancer cells. Several protein factors, including HNRNPA1, PTBP1, and Sam68, have been reported to promote PKM2 production by regulating the alternative splicing of PKM pre-mRNA [8, 15, 33]. Interestingly, we previously revealed that FEZF1-AS1 can bind and stabilize PKM2 protein to promote the proliferation and metastasis of CRC cells [9]. In this study, we revealed that LINC01852 affects aerobic glycolysis, cell proliferation and chemoresistance by inhibiting SRSF5-mediated PKM splicing. Therefore, this study revealed a novel lncRNA-mediated molecular mechanism that regulates aerobic glycolysis and cancer progression through PKM alternative splicing, highlighting the possibility that restoring LINC01852 expression may constitute a novel strategy to overcome chemoresistance and inhibit tumor growth.

Dysregulation of gene expression occur through various mechanisms, including aberrant expression of TFs, alterations in DNA methylation patterns, and dysregulated expression of noncoding RNAs. Numerous studies have shown that TFs play a critical role in the regulation of lncRNA expression. For example, ΔNp63 inhibits the transcription of NEAT1 by recruiting HDAC1 to its promoter, regulating epidermal differentiation [34]. In addition, MNX1-AS1 was found to be induced by Myc and to promote aerobic glycolysis in hepatocellular carcinoma [35]. Through bioinformatics analyses and experimental verification, we discovered that HOXD8 is downregulated in CRC and functions as a TF for LINC01852. HOXD8 has been shown to either promote or inhibit cancer progression depending on the cancer type. Some studies have suggested that HOXD8 has tumor suppressive functions in hepatocellular carcinoma and CRC [36, 37], while others have suggested that it can act as a tumor promoter in ovarian cancer and bladder cancer [38, 39]. These conflicting findings underscore the importance of studying the role of HOXD8 in specific cancer types to fully understand its potential as a therapeutic target. This study provides evidence that overexpression of HOXD8 inhibits the proliferation of CRC cells and enhances their chemosensitivity, confirming that HOXD8 acts as a tumor suppressor in CRC. This study increases the understanding of the complex role of HOXD8 in cancer, and the results may have important implications for the development of new therapeutic strategies for CRC.

## Conclusions

In summary, this study revealed that LINC01852 expression was downregulated in CRC tissues and chemoresistant CRC cells. Decreased LINC01852 expression was associated with poor clinical outcomes. Mechanistically, LINC01852 functioned as a molecular scaffold to enhance the interaction of TRIM72 with SRSF5 and facilitate TRIM72-mediated degradation of SRSF5, thus inhibiting aerobic glycolysis, tumor growth and chemoresistance. Moreover, we identified SRSF5 as a novel factor that regulates the alternative splicing of PKM, promoting PKM2 production and aerobic glycolysis. The identification of the LINC01852/TRIM72/SRSF5/PKM2 signaling axis provides new insights into the molecular mechanisms underlying drug resistance and tumor progression, identifying new therapeutic targets for CRC.

### Electronic supplementary material

Below is the link to the electronic supplementary material.

Supplementary Material 1

## Data Availability

The datasets used and/or analyzed during the current study are available from the corresponding author on reasonable request.

## Abbreviations

CRC: Colorectal cancer
LncRNAs: Long noncoding RNAs
5-FU: 5-fluorouracil
L-OHP: Oxaliplatin
ATCC: American Type Culture Collection
ASOs: Antisense oligonucleotides
qRT-PCR: Quantitative reverse transcription PCR
CCK-8: Cell Counting Kit 8
FISH: Fluorescence in situ hybridization
WB: Western blotting
IHC: Immunohistochemistry
RIP: RNA immunoprecipitation
ChIP: Chromatin immunoprecipitation
CHX: Cycloheximide
IP: Immunoprecipitation
TCGA: The Cancer Genome Atlas
SRSF5: Serine/arginine rich splicing factor 5
GSEA: Gene set enrichment analysis
OXPHOS: Oxidative phosphorylation
ESE: Exon splicing enhancer
TFs: Transcription factors
