# Supplementary material for: LINC01852 inhibits the tumorigenesis and chemoresistance in colorectal cancer by suppressing SRSF5-mediated alternative splicing of PKM
Source: Mol Cancer. 2024 Jan 24;23:23. doi: 10.1186/s12943-024-01939-7 (PMC10807094; doi:10.1186/s12943-024-01939-7)
Supplement: Supplementary file 1 — Supplementary Material 1 [file 12943_2024_1939_MOESM1_ESM.docx]

**Supplementary Materials**

**LINC01852 inhibits the tumorigenesis and chemoresistance in colorectal cancer by suppressing SRSF5-mediated alternative splicing of PKM**

Zehua Bian^1,2†^, Fan Yang^1,2†^, Peiwen Xu^1,2†^, Ge Gao^1,2^, Chunyu Yang^1,2^, Yulin Cao^2^, Surui Yao^1,2^, Xue Wang^2^, Yuan Yin^1,2^, Bojian Fei^1,2,3^, Zhaohui Huang^1,2*^

^1^ Wuxi Cancer Institute, Affiliated Hospital of Jiangnan University, Wuxi, Jiangsu, 214062, China.

^2^ Laboratory of Cancer Epigenetics, Wuxi School of Medicine, Jiangnan University, Wuxi, Jiangsu 214122, China.

^3^ Department of General Surgery, Affiliated Hospital of Jiangnan University, Wuxi, Jiangsu, 214062, China

^†^ Zehua Bian, Fan Yang and Peiwen Xu contributed equally to this work.

*Correspondence:

Zhaohui Huang, Wuxi Cancer Institute, Affiliated Hospital of Jiangnan University, 200 Hui He Road, Wuxi, 214062, China. Tel/Fax: 86-510-88682087. E-mail: zhaohuihuang@jiangnan.edu.cn.

**Supplementary figures**

**
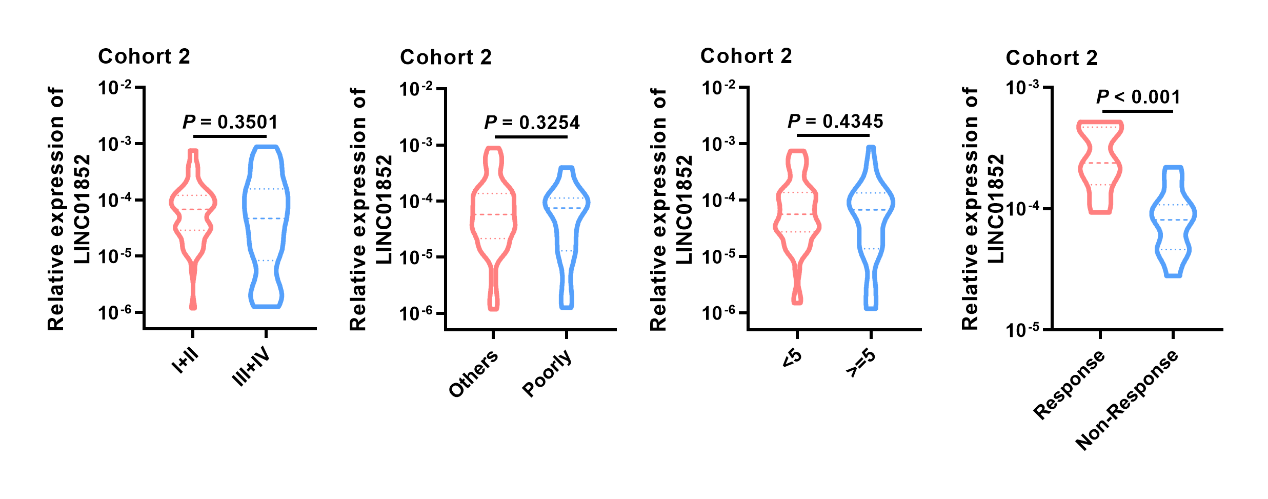
**

**Figure S1**: **Correlations between LINC01852 expression and clinicopathological characteristics in CRC patients.**


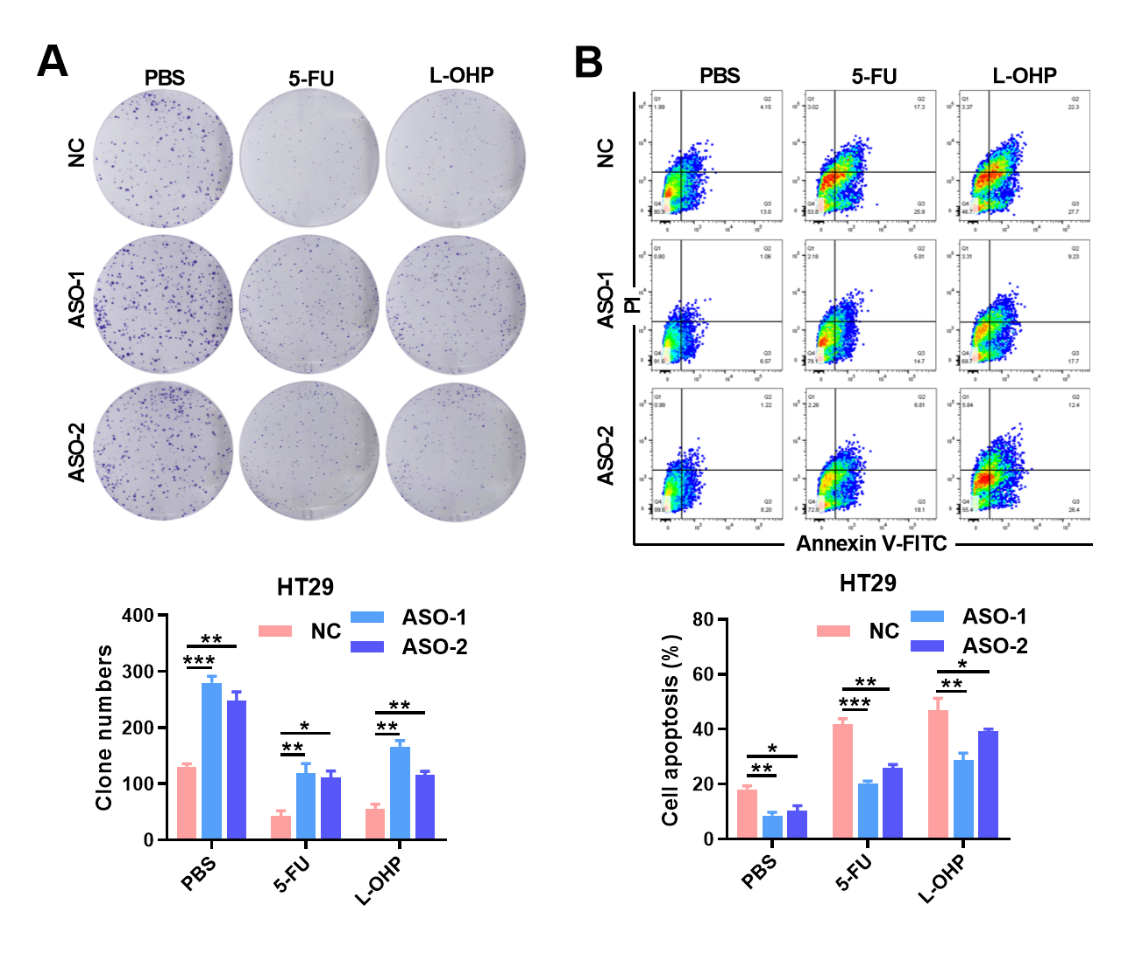


**Figure S2**: **The effects of LINC01852 knockdown on the colony formation (A) and apoptosis (B) in HT29 cells**. Cells were treated with 5-FU (10 μM) and L-OHP (4 μM) for 14 days (colony formation, A) or 48 h (apoptosis, B).


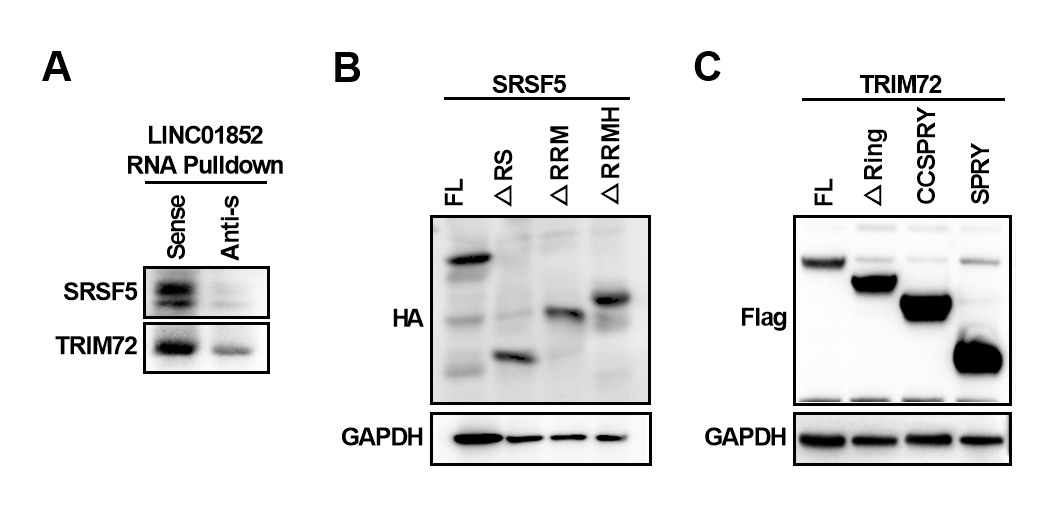


**Figure S3: Immunoblot analysis of SRSF5 and TRIM72 protein levels. (A)** Immunoblot analysis of SRSF5 and TRIM72 proteins in the protein retrieved from the LINC01852 RNA pulldown assays. **(B, C)** The protein levels of wild-type and truncated mutants of SRSF5 (B) and TRIM72 (C) were measured by WB**.**

**
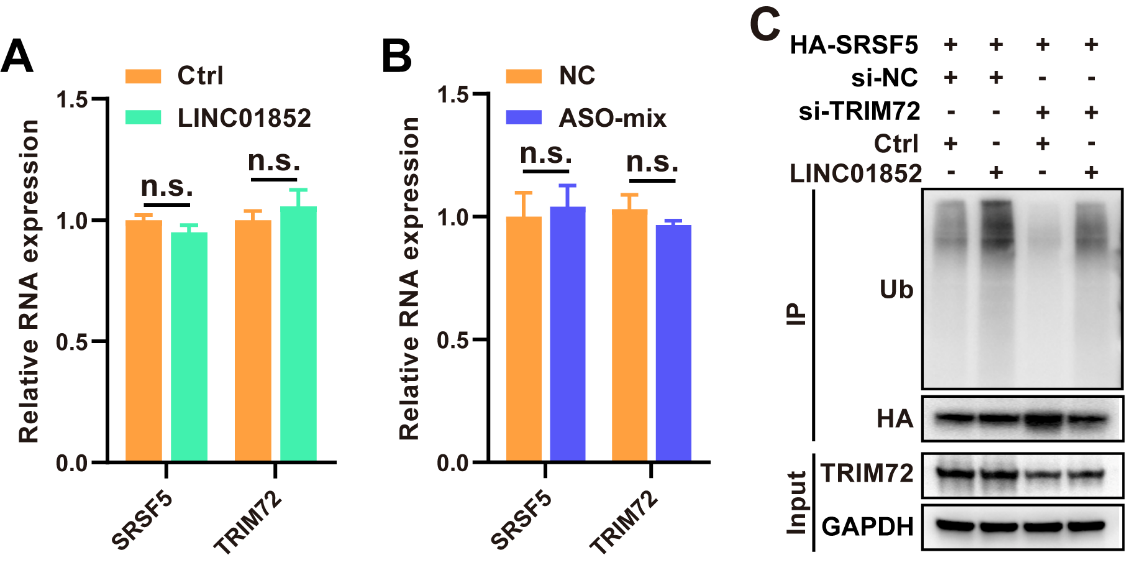
**

**Figure S4**: **LINC01852 promotes TRIM72-mediated SRSF5 ubiquitination. (A, B)** The mRNA levels of SRSF5 and TRIM72 were detected by qRT-PCR in LINC01852-overexpressing (A) and -silenced (B) CRC cells. **(C)** HEK-293T cells were transfected with the indicated vectors and si-TRIM72 for 48 h and subjected to ubiquitination assays. Prior to lysis, the cells were treated with MG132 for 6 h.


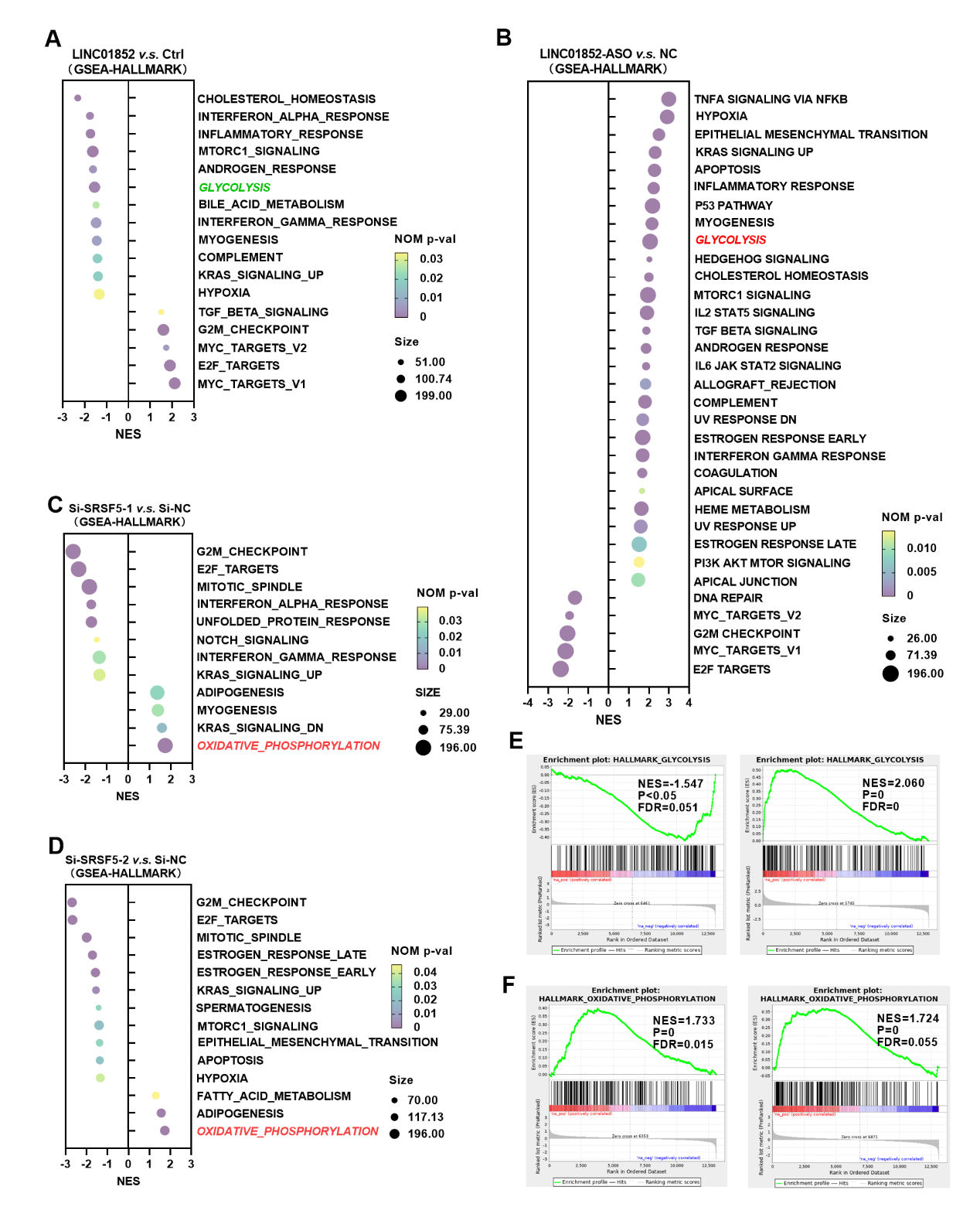


**Figure S5**: **The LINC01852/SRSF5 axis regulates energy metabolism in CRC cells.**

1. **D)** GSEA was performed to analyze the signaling pathways using differentially expressed genes in CRC cells with LINC01852 overexpression (A), LINC01852 knockdown (B), and SRSF5 knockdown (C, D) compared to their corresponding control cells. **(E, F)** High LINC01852 expression was positively related to the inhibition of glycolysis signaling (E), whereas SRSF5 was positively related to the inhibition of OXPHOS signaling (F) according to GSEA analyses.

**
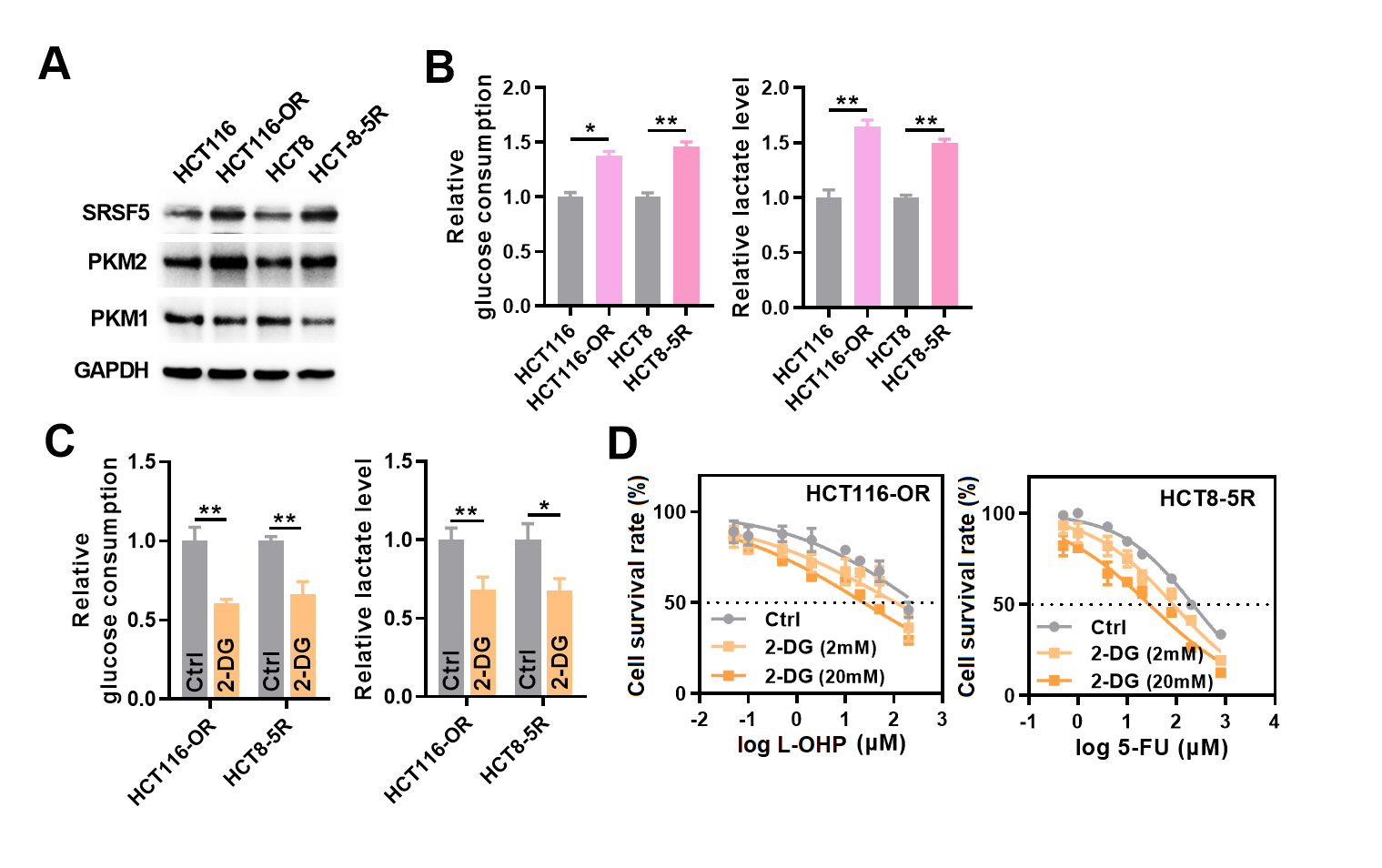
**

**Figure S6: Glycolysis promotes the chemoresistance of CRC cells. (A)** The protein levels of SRSF5, PKM2, and PKM1 were assessed via WB in HCT116-OR and HCT8-5R cells. **(B, C)** The levels of glucose uptake and lactate production were evaluated in HCT116-OR and HCT8-5R cells (B) and these cells treated with 2-DG (C). **(D)** The chemosensitivities of HCT116-OR cells to L-OHP and HCT8-5R cells to 5-FU were assessed by CCK-8 assays. ******P* < 0.05, *******P* < 0.01.

**
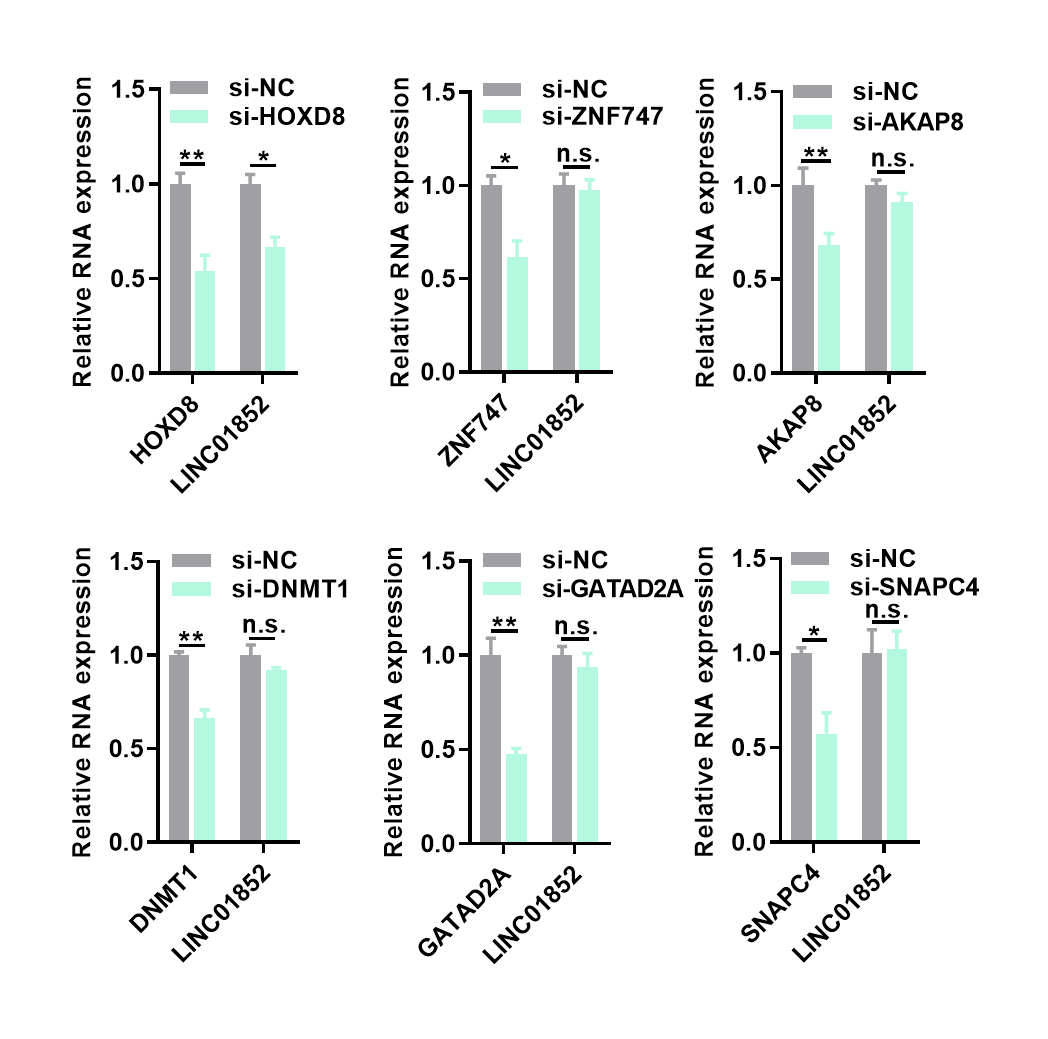
**

**Figure S7**: **The RNA levels of LINC01852 were measured by qRT-PCR in CRC cells transfected with the indicated siRNAs.**

**
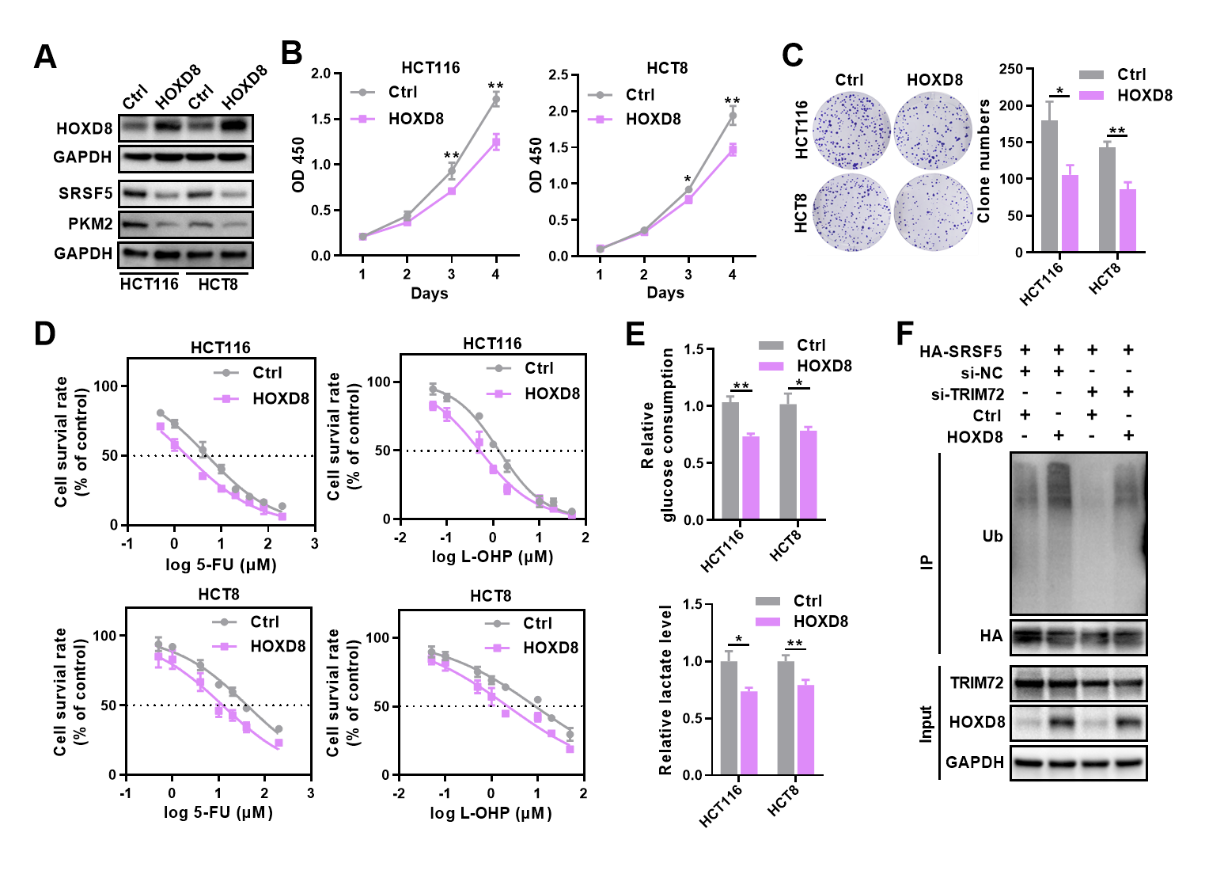
**

**Figure S8**: **HOXD8 inhibits CRC cell growth and promotes the chemosensitivity of CRC cells by promoting TRIM72-mediated ubiquitination of SRSF5. (A)** The relative protein expression of SRSF5 and PKM2 in HOXD8-overexpressing CRC cells. **(B, C)** The proliferation (B) and colony formation abilities (C) of HCT116 and HCT8 cells with HOXD8 overexpression. **(D)** The effects of HOXD8 overexpression on the chemosensitivities of CRC cells to 5-FU and L-OHP were assessed by CCK-8 assays. **(E)** The levels of glucose uptake and lactate production were measured in HOXD8-overexpressing HCT116 and HCT8 cells. **(F)** The effect of HOXD8 on TRIM72-mediated ubiquitination of SRSF5. HEK-293T cells were transfected with the indicated vectors for 48 h and subjected to ubiquitination assays. Prior to lysis, the cells were treated with MG132 for 6 h. ******P* < 0.05, *******P* < 0.01.


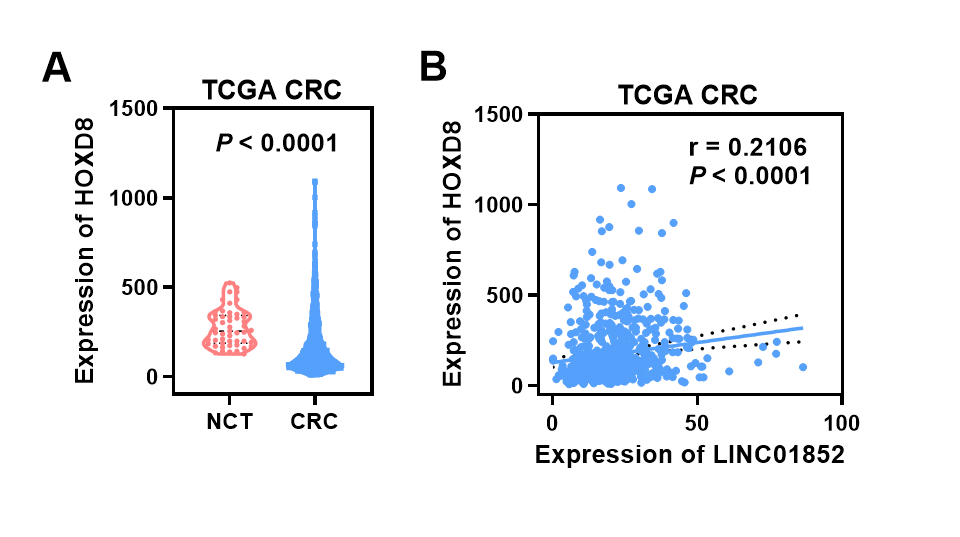


**Figure S9**: **The expression of HOXD8 (A) and its relationship with the LINC01852 expression (B) in CRC tissues from the TCGA database.**

**Supplementary Tables**

**Supplementary Table S1. Clinicopathologic features of CRC patients**

| **Characteristics** | **Colorectal cancer^a^**  **(n = 110)** | **Colorectal cancer^b^**  **(n = 177)** |
| --- | --- | --- |
| Ages (years) |  |  |
| < 60 | 63 | 75 |
| ≥ 60 | 47 | 102 |
| Gender |  |  |
| Male | 65 | 100 |
| Female | 45 | 77 |
| Tumor size (cm) |  |  |
| < 5 | 67 | 91 |
| ≥ 5 | 43 | 86 |
| Location |  |  |
| Colon | 58 | 82 |
| Rectum | 52 | 95 |
| Differentiation |  |  |
| Well and moderately | 89 | 135 |
| Poorly | 18 | 42 |
| Tumor stage |  |  |
| I+II  iiiiiiiiii  ii VCCCCCCCCCCCCCCCCCCCCCCCCCCCCCCCCCCCCCCCCCCCCCCCCCCCCCCCCCCC | 51 | 109 |
| III+IV | 59 | 68 |

^a^: LINC01852 expression data were available in 110 NCTs and 110 CRC tissues of cohort 1. ^b^: LINC01852 expression data were also available in 73 NCTs and 85 CRC tissues of cohort 2; SRSF5 expression data were available in 132 NCTs and 139 CRC tissues of cohort 2; HOXD8 expression data were available in 131 NCTs and 146 CRC tissues of cohort 2; The mRNA levels of PKM1, PKM2 and HOXD8 were available in 23 cases of cohort 2.

**Supplementary Table S2. Primer sequences**

| **Primers for real time PCR** | | |
| --- | --- | --- |
| **Primers** | **Sequence-F** | **Sequence-R** |
| LINC01852 | GCCGGGGTTCCTACTTTGAA | CAACTTCCTCCTGCAAACGC |
| β-actin | AGTGTGACGTGGACATCCGCAAAG | ATCCACATCTGCTGGAAGGTGGAC |
| U6 | CTCGCTTCGGCAGCACA | AACGCTTCACGAATTTGCGT |
| SRSF5 | AGTGGCTGTCGGGTATTCATC | CCGTCCATATCCCTTGAAGAATC |
| TRIM72 | GAGGTGGATGTTGGCGACAA | CCGAAGCTCAGGTAAAGGC |
| PKM1 | CAGCCAAAGGGGACTATCCT | GAGGCTCGCACAAGTTCTTC |
| PKM2 | ACTCGGGCTGAAGGCAGTGA | GTGGGGTCGCTGGTAATGG |
| HOXD8 | GGAAGACAAACCTACAGTCGC | TCCTGGTCAGATAGGGGTTAAAA |
| ZNF747 | GTGGGGTTTGCGTTCTTCTC | CCGCTTCTGTCGGACACTTC |
| AKAP8 | CCACAGGCGCAACCTACAG | ATGGCTCTGGGCCGTAAGA |
| DNMT1 | AGAACGGTGCTCATGCTTACA | CTCTACGGGCTTCACTTCTTG |
| GATAD2A | GAGCCCGAGAGTGAATGGG | TTCAACAACACGAGTTTTGCTTC |
| SNAPC4 | GCAATGACGAGGACGATCCC | CCTCCTGGTAGACCATGTTCAG |
| **Primers for PCR** | | |
| LINC01852-pro-PCR1 | ACGGTCCCAAACTTGAACCTAG | CTCTGAAATCCCGACGAAAGG |
| LINC01852-pro-PCR2 | TGTTTAAGCCAGAGCCTAGGAATC | ACGGAGAGCTCTAACGCCAA |
| LINC01852-pro-PCR3 | CTTGATAAAGTTAAAATTAACCCCTCTC | TCAGTATACACACAGATAGAGAGAGAG |
| PKM | CTGAAGGCAGTGATGTGGCC | ACCCGGAGGTCCACGTCCTC |
| **Sequences for gene knockdown** | | |
| LINC01852-ASO-1 | ATTCTGTATCTCCTGTGCCC | |
| LINC01852-ASO-2 | TACATTCAGGAGTGTAGGGA | |
| LINC01852-ASO-3 | GGTAGAATTCCTGGCCTCAA | |
| si-SRSF5-1 | GGATCCAAGGGATGCAGAT | |
| si-SRSF5-2 | GCACACCGACCTAAATTAA | |
| si-TRIM72-1 | CAGACTGAGTTCCTCATGA | |
| si-TRIM72-2 | GCTGCCAATTATCTCAGAT | |
| si-HOXD8 | GTCGCTTCCAAACTCTAGA | |
| si-ZNF747 | CACTGGTGGAGCACATTTA | |
| si-AKAP8 | GAGGACGAGGATGTGAAGAAGAGAA | |
| si-DNMT1 | TCCACAGATTTCTGATGAAAAAG | |
| si-GATAD2A | GAGGTTAGAAGAAGCAAAACTCG | |
| si-SNAPC4 | CAGTTAATTGAATTAATAGAAAA | |
| si-NC | TTCTCCGAACGTGTCACGT | |
| **Primers for RNA pull-down** | | |
| LINC01852-Sense-F | TAATACGACTCACTATAGGGAGAGGGGATGAATGGGGCTTTG | |
| LINC01852-Sense-R | TTAGTATCAATCCCTACACTCCTGA | |
| LINC01852-Anti-F | GGGGATGAATGGGGCTTTG | |
| LINC01852-Anti-R | TAATACGACTCACTATAGGGAGATTAGTATCAATCCCTACACTCCTGA | |
| LINC01852-(1-836nt)-R | GTGCCCACAAACACCACCAAG | |
| LINC01852-(1-609nt)-R | CCCACCACTGAGAAAATGGAAA | |
| LINC01852-(610-1278nt)-F | TAATACGACTCACTATAGGGAGAGTGGGCCGTGTCGCATA | |
| LINC01852-(206-1278nt)-F | TAATACGACTCACTATAGGGAGACCCCATTGAGAAGAACCCTTTG | |
| **Primers for cloning** | | |
| LINC01852-F | GTGAACCGTCAGATCGAATTCGGGGATGAATGGGGCTTTG | |
| LINC01852-R | TAATCCAGAGGTTGAGGATCCTTAGTATCAATCCCTACACTCCTGAATG | |
| LINC01852-pro-F-XhoI | GCGTGCTAGCCCGGGCTCGAGGAAGCCTAGCGTGAAAGAGATCA | |
| LINC01852-pro-R-HindIII | CAGTACCGGAATGCCAAGCTTGAGGCTCATTTCCATTCCTCTC | |
| LINC01852-pro-F1-XhoI | GCGTGCTAGCCCGGGCTCGAGGGGATGAACCAGCTTCTTTTGG | |
| LINC01852-pro-F2-XhoI | GCGTGCTAGCCCGGGCTCGAGCTGTTGTTGGTCTAACCTGTGGG | |
| LINC01852-pro-F3-XhoI | GCGTGCTAGCCCGGGCTCGAGCACTCAGTCACAACAAGAGGGTAAG | |
| LINC01852-pro-#1Mut-F | CTTTATGCCGTGCACTCAGTCACAACAAGAGGGT | |
| LINC01852-pro-#1Mut-R | TGAGTGCACGGCATAAAGGCTGCTCCCTAACTTTTTTT | |
| SRSF5-FL-F-BamHI | GATTACGCTTCTAGGGGATCCATGAGTGGCTGTCGGGTATTCA | |
| SRSF5-FL-R-EcoRI | AGAATCGATGATATCGAATTCTTAATTGCCACTGTCAACTGATCTG | |
| SRSF5-△RS-R-EcoRI | AGAATCGATGATATCGAATTCGTGCCTTTTGCTGCCTTCAA | |
| SRSF5-△RRM-F-BamHI | GATTACGCTTCTAGGGGATCCTCACGAGGTGGAAGAGGTAGAGG | |
| SRSF5-△RRMH-R1 | GTGCCTTTTGCTGCCTTCATTTTCTGTTCTTACAGGTGGAGCA | |
| SRSF5-△RRMH-F2 | ATGAAGGCAGCAAAAGGCACAG | |
| TRIM72-FL-F-BamHI | GATGACGACGATAAGGGATCCATGTCGGCTGCGCCCGGC | |
| TRIM72-FL-R-EcoRI | AGAATCGATGATATCGAATTCTCAGGCCTCGGCGCCTTC | |
| TRIM72-△Ring-F-BamHI | GATGACGACGATAAGGGATCCGCCCCCACGCGGCCGCAG | |
| TRIM72-CCSPRY-F-BamHI | GATGACGACGATAAGGGATCCGCCGAGGCCCACGCACGC | |
| TRIM72-SPRY-F-BamHI | GATGACGACGATAAGGGATCCCCGCAGACTGAGTTCCTCATG | |
| HOXD8-F-BamHI | GATTACGCTTCTAGGGGATCCATGAGTTCGTACTTCGTGAACCC | |
| HOXD8-R-EcoRI | AGAATCGATGATATCGAATTCTTAATTTGTCAGGCCTTCGGC | |

**Supplementary Table S3. IC50 Values (μM) of figure 6E**

|  | HCT116 | | HCT8 | |
| --- | --- | --- | --- | --- |
|  | 5-FU | L-OHP | 5-FU | L-OHP |
| Ctrl | 6.841 | 1.443 | 33.79 | 14.78 |
| SRSF5 | 17.35 | 2.967 | 88.6 | 65.36 |
| PKM2 | 19.87 | 3.586 | 93.72 | 50.03 |
| LINC | 3.156 | 0.578 | 16.53 | 4.398 |
| LINC+SRSF5 | 10.43 | 1.666 | 52.16 | 25.55 |
| LINC+PKM2 | 10.53 | 2.047 | 45.55 | 25.49 |
